# Diagnostics, taxonomy, nomenclature and distribution of perennial *Sesuvium* (Aizoaceae) in Africa

**DOI:** 10.3897/phytokeys.92.22205

**Published:** 2018-01-15

**Authors:** Alexander P. Sukhorukov, Maya V. Nilova, Andrey S. Erst, Maria Kushunina, Cláudia Baider, Filip Verloove, Marcos Salas-Pascual, Irina V. Belyaeva, Anastasiya A. Krinitsina, Peter V. Bruyns, Cornelia Klak

**Affiliations:** 1 Department of Higher Plants, Biological Faculty, Lomonosov Moscow State University, Leninskie gory 1/12, Moscow 119234, Russia; 2 Central Siberian Botanical Garden SB RAS, Zolotodolinskaya st. 101, Novosibirsk 630090, Russia; 3 Tomsk State University, Laboratory of Phylogeny and Systematics, Lenin st. 36, Tomsk 634050, Russia; 4 Department of Plant Physiology, Biological Faculty, Lomonosov Moscow State University, Leninskie gory 1/12, Moscow 119234, Russia; 5 The Mauritius Herbarium, RE Vaughan Building, Agricultural Services, Ministry of Agro-Industry and Food Security, Réduit, 80835, Mauritius; 6 Botanic Garden of Meise, Nieuwelaan 38, B-1860 Meise, Belgium; 7 Institute for Environmental Studies and Natural Resources (i-UNAT), University of Las Palmas de Gran Canaria (ULPGC), Las Palmas de Gran Canaria, Gran Canaria, Canary Islands, Spain; 8 Royal Botanic Gardens, Kew, Richmond, Surrey TW9 3AE, UK; 9 Bolus Herbarium, Department of Biological Sciences, University of Cape Town, Rhodes Gift, 7707 South Africa

**Keywords:** Africa, Aizoaceae, molecular phylogeny, new subspecies, *Sesuvium*, Sesuvieae, Sesuvioideae, taxonomy

## Abstract

The taxonomy of perennial *Sesuvium* species in Africa has been poorly investigated until now. Previously five perennial species of *Sesuvium* were recognised in Africa (*S. congense*, *S. crithmoides*, *S. mesembryanthemoides, S. portulacastrum*, and *S. sesuvioides*). Based on the differing number of stamens, *S. ayresii* is accepted here as being distinct from *S. portulacastrum*. Field observations in Angola also led the authors to conclude that *S. crystallinum* and *S. mesembryanthemoides* are conspecific with *S. crithmoides*. A new subspecies, Sesuvium portulacastrum subsp. persoonii, is described from West Africa (Cape Verde, Gambia, Guinea-Bissau, Mauritania, Senegal). The molecular phylogeny indicates the position of S. portulacastrum subsp. persoonii within the “American lineage” as a part of the *Sesuvium portulacastrum* complex which needs further studies. A diagnostic key and taxonomic notes are provided for the six perennial species of *Sesuvium* found in Africa and recognised by the authors (*S. ayresii*, *S. congense*, *S. crithmoides*, S. portulacastrum subsp. portulacastrum, S. portulacastrum subsp. persoonii, *S. verrucosum* and the facultatively short-lived *S. sesuvioides*). The distribution of *S. crithmoides*, previously considered to be endemic to Angola, is now confirmed for the seashores of Republic of Congo and DR Congo. The American species *S. verrucosum* is reported for the first time for Africa (the Macaronesian islands: Cape Verde and the Canaries). It is locally naturalised in Gran Canaria, being a potentially invasive species. These findings as well as new records of *S. verrucosum* from Asia and the Pacific Islands confirm its proneness to transcontinental introduction. Lectotypes of *S. brevifolium*, *S. crithmoides*, *S. crystallinum* and *S. mesembryanthemoides* are selected. The seed micromorphology and anatomy of the perennial African species is studied. Compared to the seeds of some annual African *Sesuvium* investigated earlier, those of perennial species are smooth or slightly alveolate. The aril is one-layered and parenchymatous in all species and usually tightly covers the seed. The aril detachments from the seed coat that form a white stripe near the cotyledon area easily distinguish *S. verrucosum* from other species under study.

## Introduction

*Sesuvium* L. is one of the most widespread genera of Aizoaceae occuring in many subtropical and tropical regions of the world (Bohley et al. 2017). The perennial *Sesuvium* species often form mono- or oligodominant plant communities in coastal areas (e.g. Oliver 1871, Sauer 1982, Nellis 1994). *Sesuvium portulacastrum* (L.) L. is considered to be the species with the widest distribution pattern on all continents compared to the other representatives of the genus (Bogle 1970, Lonard and Judd 1997, Bohley et al. 2015). During the last decades the number of recognised species changed from eight (Bogle 1970) to twelve (Hartmann 1993) and reached fourteen after the inclusion of three American species of *Cypselea* Turpin (Bohley et al. 2017).

In its current circumscription, *Sesuvium* includes perennial or annual herbs with prostrate or ascending, often rooting at the nodes, glabrous or vesiculose stems (additionally with stout warts when dry; Sukhorukov et al. 2017); opposite, more or less succulent leaves with short or hardly visible petioles, which bear two semi-amplexicaulous, membranous or hyaline, entire or fimbriate, marginally concrescent stipules; axillary, bracteolate, pedicellate or sessile flowers; five, bi-coloured (green dorsally and pink or white ventrally) perianth lobes; five to numerous pink stamens; ovary consisting of two to five carpels; circumscissile capsule with the central column bearing 5–50 black or reddish, smooth or diversely sculptured seeds completely or partially covered with thin and hyaline aril.

*Sesuvium* is the type genus of subfamily Sesuvioideae (Lindley 1853, as “Sesuvieae”) which is characterised by stipulate or stipule-like leaf margins; bracteolate, perigynous flowers; externally sepaloid and internally petaloid perianth with the segments mostly apiculate on the back towards the apex, circumscissile capsule and seed usually covered by an aril (Hartmann 1993). Sesuvioideae is sister to all other Aizoaceae (Klak et al. 2003). Two major subclades were recognised within this subfamily: Sesuvioideae s.str. and *Tribulocarpus* S.Moore (Klak et al. 2003, Thulin et al. 2012). A recent study found the monotypic *Anisostigma* Schinz to be closely related to *Tribulocarpus* (Klak et al. 2017a), which together are now recognised as the tribe Anisostigmateae Klak (Klak et al. 2017b). The Sesuvioideae therefore now consists of two tribes, the Anisostigmateae (two genera) and the Sesuvieae comprising *Sesuvium* (including *Cypselea*), *Trianthema* L. and *Zaleya* Burm.f. *Sesuvium* is divided into two subclades, the American lineage with C_3_ photosynthesis (*S. portulacastrum*, *S. verrucosum* Raf., *S. maritimum* (Walter) Britton, Sterns & Poggenb., as well as the species formerly included in *Cypselea*) and the African lineage comprising the native African species with a C_4_ photosynthetic pathway (Bohley et al. 2017). The species of each lineage are characterised by several types of leaf anatomy and are distinguished by the shape of the epidermal cells and by the mesophyll structure (Bohley et al. 2015). In the previous paper (Sukhorukov et al. 2017), the annual species of *Sesuvium* in Africa were revised. Instead of one (e.g. Jeffrey 1961, Hartmann 2002) or two (Bohley et al. 2017) species, four native species were accepted (*S. digynum* Welw., *S. hydaspicum* (Edgew.) Gonç., *S. nyasicum* (Baker) Gonç. and *S. sesuvioides* (Fenzl) Verd.) based on morphological and carpological characters. A new taxonomic treatment of the entire genus (Bohley et al. 2017) suggested the presence of five perennial species of *Sesuvium* in Africa: *S. congense* Welw., *S. crithmoides* Welw., *S. mesembryanthemoides* Wawra, *S. portulacastrum* and *S. sesuvioides* (also as a perennial species). All perennial taxa usually grow on the seashores of tropical Africa. One of them – *S. portulacastrum* – is considered to be a widespread species on the continent (Hutchinson and Dalziel 1927, Jeffrey 1961, Lonard and Judd 1997), whereas three others – *S. congense*, *S. crithmoides* and *S. mesembryanthemoides* – have been documented for Angola only (Welwitsch 1859, Oliver 1871, Bohley et al. 2017). However, Bohley et al. (2017) acknowledged that some of their taxonomic conclusions have been tentative and that further more detailed studies would be required to establish species limits within *Sesuvium* (e.g., *S. crithmoides*). Hereby, the results of such a study are published.

The authors’ own field investigations, revision of relevant herbarium material and further taxonomic studies revealed a greater diversity of the perennial *Sesuvium* in Africa in contrast to the latest revision of the genus worldwide (Bohley et al. 2017). Additionally, the fine seed traits (micromorphology and anatomy) of perennial *Sesuvium* have been studied for the first time and some new samples have been added to the molecular analysis. Based on this, an improved taxonomy and phylogeny have been presented and the distribution of the perennial *Sesuvium* in Africa has been discussed.

## Methods

### Field studies and revision of the herbarium material

Field investigations were performed by the first author (AS) in Sal and Boa Vista Islands, Republic of Cape Verde (August 2015, January and September 2016) and in Namibia (March 2017); by Cláudia Baider in Mauritius (2017); by Marcos Salas-Pascual (2016) and Filip Verloove (March–April 2017) in the Canary Islands (Spain) and by Cornelia Klak and Peter Bruyns in Angola (December 2016–January 2017). Additionally, the first author (AS) examined herbarium specimens in the herbaria B (on loan in Mainz, Germany), BM, BR, E, G, K, L (incl. U and WAG, but the African material in WAG was on loan), LE, LY, LYJB, M, MHA, MSB, MW, P, WIND; Filip Verloove identified the material in LPA; Cláudia Baider revised the specimens in MAU and Cornelia Klak in BOL, LUBA and PRE. In addition, some material of *Sesuvium portulacastrum* (leaves and seeds) collected by AS in Grenada (Lesser Antilles, Caribbean Islands) and Israel (as a cultivated plant in the Dead Sea area) was also used for anatomical and molecular studies.

To assess the conservation status of each taxon as per the IUCN Red List, past and present distribution data from herbarium specimens were collated. When the original specimen label did not give the precise location, a geographical point centred in the locality of the collection cited was used. This information was then assessed based on available ecological data or review of threats to allow insights into understanding the current population and distribution trends useful in defining the IUCN Red List Categories and Criteria (IUCN 2017). The extent of occurrence (EOO) and area of occurrence (AOO) were calculated using GeoCAT ver. β, with a cell of 2 × 2 km^2^ (Bachman et al. 2011). These assessements were not sent to the respective SSC IUCN groups prior to the publication of this article.

### Leaf anatomy

The leaves of Sesuvium portulacastrum subsp. persoonii were collected by AS in August 2015 in Cape Verde (Sal Island, near Santa Maria village) and soaked in a 70% alcohol solution. The sections were made by hand and stained with 0.2% aqueous toluidin blue. For the description of the leaf anatomy, the terminology by Bohley et al. (2015) was followed. The leaf structure was photographed with a Nikon DS-Vi1 camera (Nikon Corporation, Japan) at the Department of Higher Plants, Lomonosov Moscow State University.

### Seed morphology and anatomy

Seed micromorphology was observed using a scanning electron microscope (SEM) JSM–6380 (JEOL Ltd., Japan) at 15 kV after sputtercoating with gold-palladium in the laboratory of Electron Microscopy at Lomonosov Moscow State University. No dehydration of the seeds was required prior to SEM observation due to the absence of soft tissues (e.g. papillae or trichomes) on their surface.

The cross-sections of the seeds were prepared using a rotary microtome Microm HM 355S (Thermo Fisher Scientific, USA) and photographed with a Nikon DS-Vi1 camera (Nikon Corporation, Japan) at the Department of Higher Plants, Lomonosov Moscow State University. Before sectioning, the seeds were soaked in water:alcohol:glycerin (1:1:1) solution, dehydrated in ethanol dilution series and embedded in the Technovit 7100 resin (Heraeus Kulzer, Germany).

The list of specimens used for SEM (perennial species) and anatomical investigations (both annual and perennial taxa) is given below. For seed morphology of the annual *Sesuvium* taxa, see Sukhorukov et al. (2017).

*Sesuvium ayresii* Marais: Ilot Marianne, 18 Jan 1975, *Lorence 1059* (K);

*S. congense* Welw.: Angola, Lengue, 19 Dec 1932, *Grossweiler 9715* (BM); Angola, Porto Alexandre, Aug 1937, *H. Humbert 16375* (BM);

*S. crithmoides* Welw.: Angola, Kabinda, 30 Nov 1957, *Lebrun 111905* (K); Angola, Luanda, 12 Jun 1858, *Welwitsch 2386* (BM000839897) as *S. crystallinum* Welw.;

*S. digynum* Welw.: Angola, Mossamedes [Namibe], 8 May 1963, *A. De Menezes 409* (K);

*S. hydaspicum* (Edgew.) Gonç.: Saudi Arabia, South Hijag, 29 Mar 1979, *J.S. Collenette 1153* (K);

*S. nyasicum* (Baker) Gonç.: [Malawi] Nyassa [Lake Malawi], Monkey Bay, Aug 1896, *A. Whyte s.n*. (K000076291); Namibia, Hardap Region, 2 Mar 2017, *A. Sukhorukov s.n*. (MW);

S. portulacastrum (L.) L. subsp. portulacastrum: 1) [Mexico, Colima State] Revillagigedo Island, 23 Mar 1932, *J.T. Howell 8353* (K); 2) Grenada, St.-George’s, 1 Dec 2016, *A. Sukhorukov 684* (MW);

S. portulacastrum (L.) L. subsp. persoonii Sukhor.: Senegal, St. Louis, 23 Jul 1960, *J.D. Kesby 20* (K); Cape Verde, Sal Island, Santa Maria, 30 Aug 2015, *A. Sukhorukov 59* (MW);

*S. sesuvioides* (Fenzl) Verd.: Angola, Mossamedes [Namibe], Praia Amelia, 28 Dec 1955, *E.J. Mendes 1172* (BM);

*S. verrucosum* Raf.: USA, California, San Joaquin co., 4 Jul 1934, *E. Lee 963* (H1283635); USA, Nevada, Pershing co., 31 Aug 2000, *A. Thielim 13396* (M).

### DNA extraction and PCR

Total DNA was extracted from silica gel-dried or fresh material of *S. portulacastrum* (collected in Israel and Grenada), S. portulacastrum subsp. persoonii (Cape Verde) and *S. nyasicum* (Namibia). The DNA from fresh material was extracted according to Krinitsina et al. (2015) and that from dry leaves was extracted using DiamondDNA Plant kit (DiamondDNA, Russia) with further purification using AMPure Beads (Beckman Coulter, USA) (for details see Krinitsina et al. 2015).

The nuclear ITS region (internal transcribed spacer 1, 5.8S ribosomal RNA gene and internal transcribed spacer 2) and three plastid regions (*rps16* gene intron, *atpB-rbcL* intergenic spacer, *trnL*-*trnF* intergenic spacer) were sequenced. PCR amplifications were carried out in a Thermal Cycler T100 (Bio-Rad, USA) using primers and cycler programmes listed in Table 1. The reaction mix (25 μl) contained 10 ng of DNA, 1 μM of each primer, 200 μM of each dNTP and 0.5 U hot start TagF polymerase (AmpliSens, InterLabService, Russia). PCR products were checked on 1.2% agarose gels and purified using AMPure Beads (Beckman Coulter, USA) according to the manufacturer’s protocol. AMPure Beads suspension was mixed with a solution containing PCR-product at the ratio 1.2:1 (for ITS and *atpB-rbcL* primer pairs) or 1.4:1 (for all other primer pairs). The sequencing was performed at Genome centre, Engelhardt Institute of Molecular Biology (Moscow, Russia) on Applied Biosystems 3730 DNA Analyser using ABI PRISM® BigDye™ Terminator v.3.1 Cycle Sequencing Kit.

**Table 1.** Primers and cycler programmes used for the molecular analysis.

Marker	Primer sequences and combination	Reference	Cycler programmer
ITS	ITS5 5’-GGA AGT AAA AGT CGT AAC AAG G-3’ / ITS4 5’-TCC TCC GCT TAT TGA TAT GC-3’	White et al. 1990	95 °C for 15 min, 5 cycles of amplification (95 °C for 30 s, 53 °C–49 °C for 1 min (–1 °C per cycle), 72 °C for 1 min), 30 cycles of amplification (95 °C for 15 s, 50 °C for 30 s, 72 °C for 40 s), 72 °C for 5 min
*rps16-intron*	rps16 F 5’-GTG GTA GAA AGC AAC GTG CGA CTT-3’ / rps 16 intr R 5’-CTT GTT CCG GAA TCC TTT ATC-3’	rps16 F and rps16 R (Oxelman et al. 1997); rps16 int F and rps 16 intr R (Bohley et al. 2015)	95 °C for 15 min, 35 cycles of amplification (95 °C for 1 min, 50 °C–65 °C (increasing by 0.3 °C per cycle) for 1 min, 72 °C for 4 min), 72 °C for 5 min
	rps16 int F 5’-GTA TGT TGC TGC CAT TTT TGA AAG G-3’ / rps16 R 5’-TCG GGA TCG AAC ATC AAT TGC AAC-3’		
*atpB-rbcL* spacer	atpB-rbcL F 5’-GAA GTA GTA GGA TTG ATT CTC-3’ / atpB-rbcL R 5’-CAA CAC TTG CTT TAG TCT CTG-3’	Golenberg et al. 1993	95 °C for 15 min, 35 cycles of amplification (95 °C for 20 s, 56 °C for 30 s, 72 °C for 60 s), 95 °C for 20 s, 56 °C for 80 s, 72 °C for 8 min
*trnL-F*	Tab C 5’-CGA AAT CGG TAG ACG CTA CG-3’ / Tab D 5’-GGG GAT AGA GGG ACT TGA AC-3’	Tab C, Tab D and Tab F (Taberlet et al. 1991); trnL-F inter F (Bohley et al. 2015)	95 °C for 15 min, 35 cycles of amplification (95 °C for 1 min, 50 °C–65 °C (increasing by 0.3 °C per cycle) for 1 min, 72 °C for 4 min), 72 °C for 5 min
	trnL-F inter F 5’-GGA CGA GAA TGA AGA TAG ACT C-3’ / Tab F 5’-ATI’ TGA ACT GGT GAC ACG AG-3’		

### Sequence alignment and phylogenetic reconstruction

The raw forward and reverse sequences were checked and combined in BioEdit sequence alignment editor v. 7.0.5.3 (Hall 1999). The sequences were aligned using Muscle algorithm and MEGA6.0 software package (www.megasoftware.net; see Tamura et al. 2013). Two data sets were assembled: (1) consisting of three chloroplast markers and (2) the nuclear (ITS) gene region. These data sets were first analysed separately and then in combination using the Maximum Likelihood (ML) method in MEGA 6.0 (Tamura et al. 2013) and Bayesian Inference (BI) in BEAST (Bouckaert et al. 2014). A bootstrapping of 1,000 replicates for ML analysis was processed to estimate the confidence probabilities on each branch of the phylogenetic trees constructed. An initial tree (ML) for the heuristic search was obtained by applying the Neighbour-Joining method to a matrix of pairwise distances estimated using the Maximum Composite Likelihood approach. All positions containing gaps were treated as missing data. Bayesian analyses were run for 20,000,000 generations with four MCMC chains in two independent runs. The first 2,000,000 samples from each run were discarded as burn-in. Convergence was assessed by comparing standard deviation of split frequencies between different runs (MCMC Trace Analysis Tool (Tracer) version v1.6.0; Rambaut et al. 2014). For ML and BI analyses, optimal models of molecular evolution for combined matrices were identified using jModelTest2 (Darriba et al. 2012) (optimal model is GTR + G). Voucher information and GenBank accession numbers are listed in Table 2.

**Table 2.** Voucher information and GenBank accession numbers for perennial *Sesuvium* species and outgroups included in the phylogenetic analysis. The newly sequenced samples are highlighted in bold.

**Species**	**Voucher information (country, year, herbarium acronym and number)**	**GenBank accession number**			
		***rps 16* intron**	***atpB-rbcL* intergenic spacer**	***trnL-trnF* intergenic spacer**	**ITS**
*Sesuvium congense*	Angola, 2009 (PRE849008.8)	KJ848244.1	KJ848148.1	KJ848291.1	KJ848196.1
*S. crithmoides*	Angola, 2009, (PRE849042.0)	KJ848247.1	KJ848151.1	KJ848294.1	KJ848199.1
*S. humifusum* (ex-*Cypselea humifusa*)	USA (MJG014141)	KJ848241.1	KJ848145.1	KJ848288.1	KJ848193.1
*S. hydaspicum*	Burkina Faso, 1996 (MO055896)	KJ848230.1	KJ848136.1	KJ848277.1	KJ848181.1
*S. hydaspicum*	Burkina Faso, *Madsen 5264* (S)	–	–	–	AJ937561.1
*S. maritimum*	Mexico, 1999 (BRIT)	–	–	–	KJ848178.1
*S. maritimum*	USA, Louisiana, *Thomas et al. 103258* (NY)	–	–	–	AJ937562.1
*S. maritimum*	USA, Texas, *Walker 1673* (NY)	–	–	–	AJ937563.1
*S. maritimum*	USA, [North Carolina], 1998 (BRIT)	KJ848228.1	KJ848134.1	KJ848275.1	KJ848179.1
*Sesuvium* sp.	Namibia, 1996 (MO5667010)	–	–	–	KJ848190.1
*Sesuvium* sp.	Angola, 2009 (PRE849020)	KJ848246.1	KJ848150.1	KJ848293.1	KJ848198.1
***S. nyasicum***	**Namibia, 2017, *Sukhorukov s.n*. (MW)**	**MG209774**	**MG209769**	**MG209777**	**MG495932**
***S. portulacastrum***	**Israel, Dead Sea, *Sukhorukov s.n*. (MW)**	**MG209775**	**MG209772**	**MG762002**	**MG461526**
***S. portulacastrum***	**Grenada, St.-George’s, 2016, *Sukhorukov 684* (MW)**	**MG209776**	**MG209771**	**MG209779**	–
*S. portulacastrum*	Morocco, 2012 (MJG014142)	KJ848232.1	KJ848138.1	KJ848279.1	KJ848183.1
*S. portulacastrum*	Saint Kitts and Nevis, 1994 (MO5158713)	KJ848236.1	KJ848141.1	KJ848284.1	KJ848188.1
*S. portulacastrum*	Mexico, 2010 (MJG014143)	KJ848240.1	KJ848144.1	KJ848287.1	KJ848192.1
*S. portulacastrum*	USA, Florida, 2013 (MJG014144)	KJ848243.1	KJ848147.1	KJ848290.1	KJ848195.1
*S. portulacastrum*	Taiwan, 2003 (MO6268738)	–	–	–	KJ848185.1
*S. portulacastrum*	Venezuela (ex cult., *Thiede s.n*. (HBG))	–	–	–	AJ577758.1
*S. portulacastrum*	Bolivia, 1998 (MO5903990)	–	–	–	KJ848184.1
*S. portulacastrum*	India, anonym (RK402)	–	–	–	FJ784241.1
*S. portulacastrum*	India, [without herbarium voucher]	–	–	–	KC185421.1
*S. portulacastrum*	India, anonym (AUFMS260)	–	–	–	KF848298.1
**S. portulacastrum subsp. persoonii**	**Cape Verde, Sal Island, *Sukhorukov 59* (MW)**	**MG209773**	**MG209770**	**MG209778**	**MG495933**
*S. sesuvioides*	Namibia, 1988 (HBG910260)	KJ848231.1	KJ848137.1	KJ848278.1	KJ848182.1
*S. sesuvioides*	Angola, 2009 (PRE8499750)	KJ848245.1	KJ848149.1	KJ848292.1	KJ848197.1
*S. sesuvioides*	Namibia, *Van Slageren & Brand MSJB020* (WAG)	–	–	–	AJ937583.1
*S. verrucosum*	USA, [California], 1999 (BRIT)	KJ848229.1	KJ848135.1	KJ848276.1	KJ848180.1
*S. verrucosum*	Mexico, 2004 (MEXU 1237208)	KJ848237.1	KJ848142.1	KJ848285.1	KJ848189.1
*S. verrucosum*	USA, [Nevada], 2013 (MJG014145)	KJ848242.1	KJ848146.1	KJ848289.1	KJ848194.1
*S. verrucosum*	Saudi Arabia, *Fayed S.n*. (UBT)	–	–	–	AJ937564.1
*S. verrucosum*	United Arab Emirates, Dubai, *Hartmann & Hartmann 34761* (HBG)	–	–	–	HE585045.1
*S. verrucosum*	Mexico, 1998 (MEXU1231179)	–	–	–	KJ848191.1
*Portulaca oleracea* (outgroup)	South Korea, Jeollanam-do prov., Gisan-ri, 2013 (JKTM1000081)	–	–	–	KM051437.1
*Phytolacca dioica* (outgroup)	Garden material, South Africa, 2002, *Klak 988* (BOL)	AJ532733.1	AJ532612.1	KM261955.1	–

## Results and discussion

### Leaf anatomy

**Figure 1.**
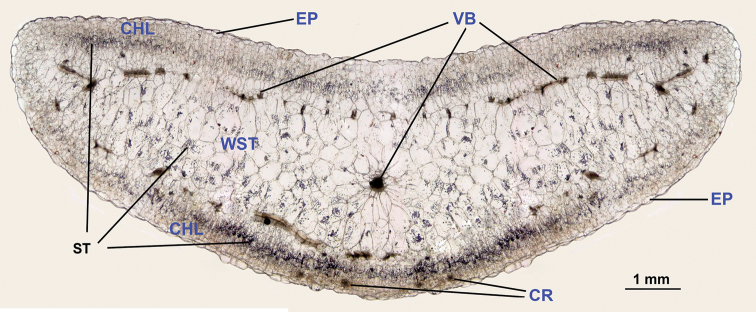
Leaf anatomy of S. portulacastrum subsp. persoonii. Abbreviations: **CHL** chlorenchyma **EP** epidermis **CR** crystals (druses) **ST** starch grains in the palisade cells **VB** vascular bundles **WST** water storage tissue. Scale bar: 1 mm.

The leaf anatomy of a new subspecies S. portulacastrum subsp. persoonii (Fig. 1) was investigated.

*Description*: Leaves terete, of lenticular shape in cross-sections, very succulent, leaf thickness ~4.2 mm; epidermis of the adult leaves mamillate (with slightly convex outer cell walls); hypodermis absent; mesophyll with palisade and water storage cells; palisade cells forming chlorophyll-containing tissue arranged in 3–7 layers below the epidermis (~0.6–0.7 mm from each leaf side), with abundant druses; the cells of innermost palisade layer and adjacent cells of water storage tissue with abundant starch grains (looking like dark stripes: Fig. 1); water storage cells arranged in numerous layers; one or rarely two main vascular bundles in the centre of the leaf are present, with numerous lateral vascular bundles.

The anatomical structure of the leaves of S. portulacastrum subsp. persoonii is similar to that of *S. portulacastrum* (type subspecies) described by Bohley et al. (2015). The difference between the “*Tribulocarpus* type” (e.g. *Sesuvium maritimum*, *S. verrucosum*, some individuals of *portulacastrum* with papillate leaves) and the “*Sesuvium portulacastrum* type” (glabrous forms of *S. portulacastrum* and *S. maritimum*) appears to lie only in the presence or absence of papillae (bladder cells) on the leaf epidermis (Bohley et al. 2015). Therefore, the authors propose to unite these two types of leaf anatomy into the “*Sesuvium portulacastrum* type”.

### Flower, fruit and seed characters

The position of the ovary in *Sesuvium* is considered superior (e.g. Jeffrey 1961, Adamson 1962, Bogle 1970, Gonçalves 1995) or semi-inferior (Ferren 2003). Sometimes the flowers are described as perigynous (Hartmann 2002, Hassan et al. 2005b, Bohley et al. 2017), but this term does not describe the insertion of the ovary as compared to other floral parts. In fact, the connate part of the tepals forms a true hypanthium, with concrescence of the lower parts of the filaments with the inner surface of the flower cup. The insertion of the stamens seems to be near the top of the hypanthium. However, the ovary itself is situated above the point where the other floral parts are inserted and it should therefore be considered superior as in other Sesuvioideae (Bogle 1970). The perigynous flowers and superior position of the ovary are very characteristic traits for the Sesuvioideae as the basal-most lineage within the Aizoaceae.

The fruit in *Sesuvium* is a circumscissile capsule. The capsule is usually shorter than or rarely almost equal to the tepals, especially in some annual species. The reproductive diaspore type is a seed. The mode of seed dispersal in *Sesuvium* has not yet been investigated, but it was suggested that the seeds might be dispersed by water (Marais 1990, Tomlinson 2016). Taking into account the coastal habitats where almost all perennial species of the genus are found, this assumption seems to be reasonable. All plant parts of perennial *Sesuvium* in coastal areas are grazed by cattle (Burkill 1985) and thus endozoochory may also be an important mode for dispersal. The thick seed coat protects the embryo against long-lasting water impact or digestion, as in many other species of Caryophyllales with similar dispersal facilities requiring embryo protection (e.g. Netolitzky 1926, Sukhorukov 2008, Sukhorukov and Zhang 2013, Sukhorukov et al. 2015).

**Figure 2.**
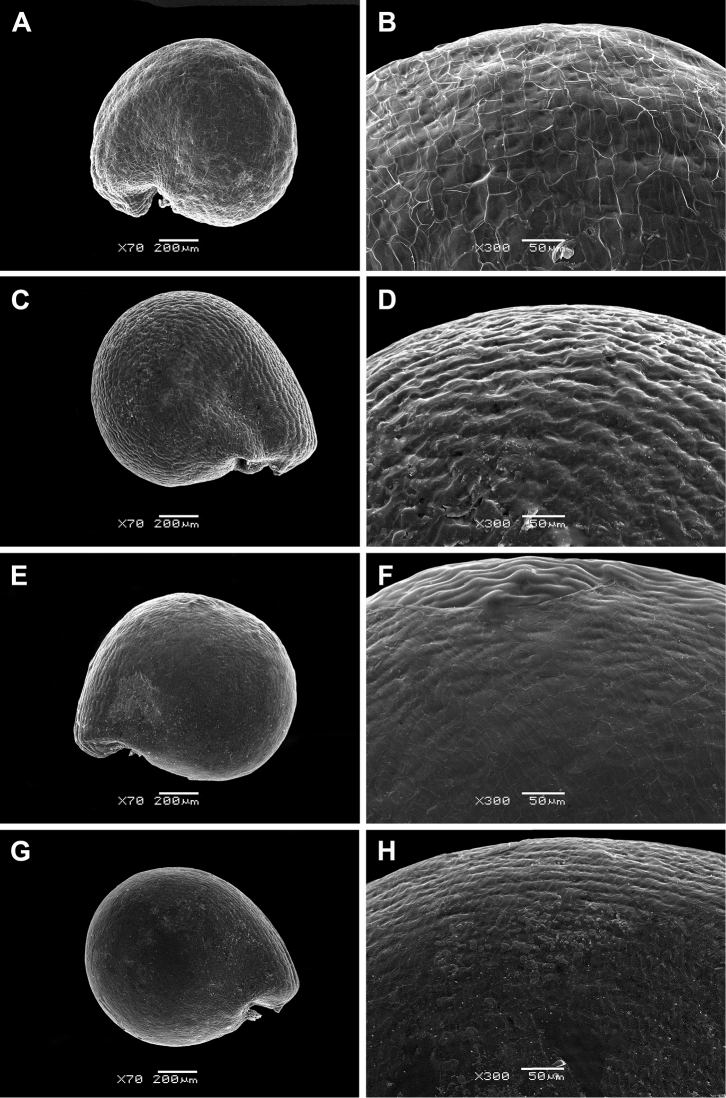
SEM micrographs of *Sesuvium* seeds (covered with an aril). **A, B** *S. ayresii* **C, D** *S. congense* **E, F** *S. crithmoides* **G, H** *S. crystallinum* (now merged with *S. crithmoides*). Magnification: **A, C, E, G**: 70×; **B, D, F, H** : 300×.

**Figure 3.**
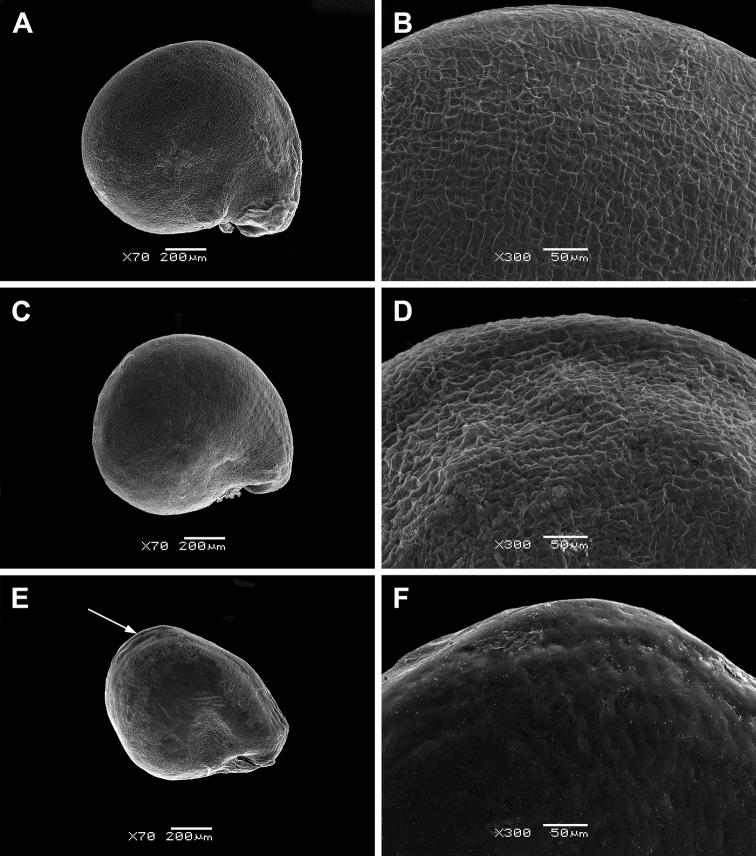
SEM micrographs of *Sesuvium* seeds (covered with an aril). **A, B** S. portulacastrum subsp. persoonii **C, D** S. portulacastrum subsp. portulacastrum **E, F** *S. verrucosum*. Magnification: **A, C, E**: 70×; **B, D, F**: 300×. Arrow on image E indicates the detachment of the aril from the seed coat forming a distinctive fold in the cotyledon area.

The seeds of all perennial *Sesuvium* under study are roundish, 0.9–1.1 mm in diameter and slightly flattened (Figs 2 and 3). The aril is one-layered, whitish, ca. 1–2 µm thick in cross-section and consists of thin-walled cells. It tightly adheres to the seed coat. However, *S. verrucosum* is distinguished by the small detachments of the aril from the seed coat forming a distinctive fold in the cotyledon area (Fig. 3E). The aril usually covers the seed completely (Figs 2, 3A–D), but in some seeds of *S. portulacastrum*, it is only partially present. An aril covering up to half the seed surface is not common in *S. portulacastrum* (or any other *Sesuvium*) mentioned by Hassan et al. (2005a). The presence of a tiny aril apparently does not provide any protective function and its role in seed dispersal or germination is unclear.

**Figure 4.**
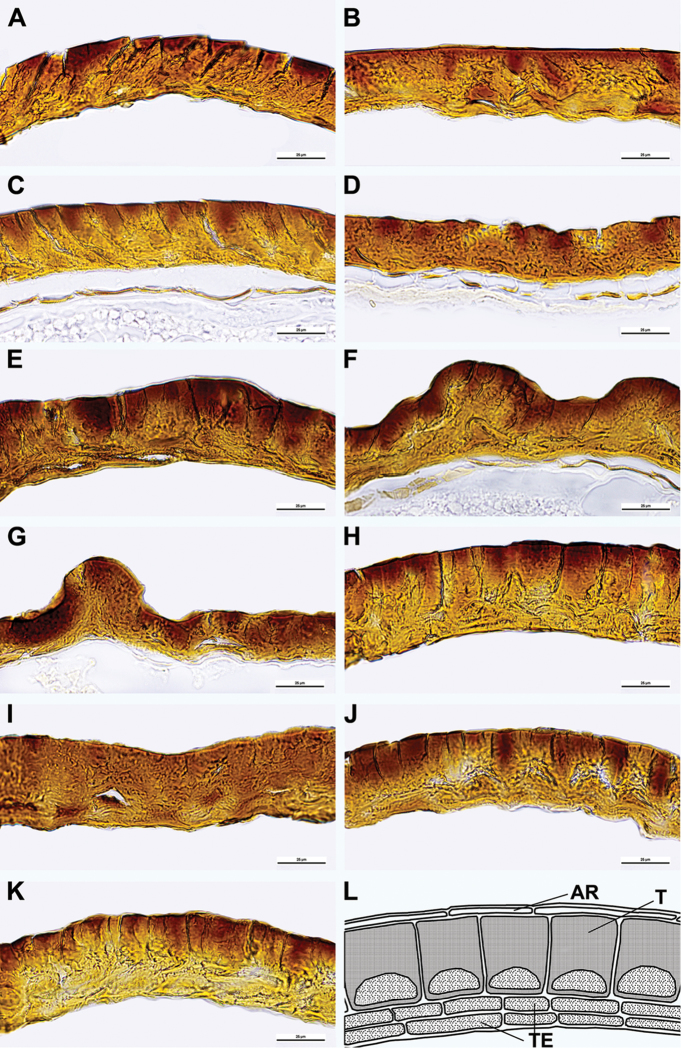
Seed anatomy of annual and perennial *Sesuvium* species in Africa: **A** *S. ayresii* **B** *S. congense* **C** *S. crithmoides* **D** *S. crystallinum* (now merged with *S. crithmoides*) **E** *S. digynum* **F** *S. hydaspicum* **G** *S. nyasicum* **H** S. portulacastrum subsp. persoonii **I** S. portulacastrum subsp. portulacastrum **J** *S. sesuvioides* **K** *S. verrucosum* **L** schematic drawing of the seed structure. Scale bar: 25 µm. Abbreviations (image **L**): **AR** seed aril; **T** testa; **TE** tegmen.

The seed coat of perennial *Sesuvium* is smooth or slightly wavy, often with small, radially elongated striae. Hardly noticeable pits were found only in *S. verrucosum* (Fig. 3F). In cross-section, the testal layer is much thicker than the 1–3 endotegmal layers. In almost all species, the testa thickness ranges from (25–)30 to 50 µm (Fig. 4), but the testa of a *S. portulacastrum* specimen from Grenada studied for comparison was found to measure between 70 and 80 µm. The outer periclinal wall of the testa cells is clearly thicker than the inner periclinal wall and the protoplast is usually clearly visible. The walls and protoplast of the testa cells are completely filled with tannins, especially the external areas of the outer cell walls, which appear dark brown. The “stalactites” in the outer cell walls are not prominent in comparison to other representatives of the core Caryophyllales (Takhtajan 1991, Sukhorukov and Zhang 2013, Sukhorukov et al. 2015). The thickness of the tegmen layers is 2–8 µm (each layer has an average thickness of 3 µm). The embryo is annular and the perisperm is copious.

There are no significant differences in seed structure between perennial and annual *Sesuvium* species growing in Africa. However, the seed-coat testa of some annual African *Sesuvium* (*S. hydaspicum* and especially *S. nyasicum*) has wrinkle- or ridge-like outgrowths (Sukhorukov et al. 2017). In all other species, the seeds are smooth, except for the annual North American *Sesuvium trianthemoides* Correll with rugose seed ornamentation (Correll 1966). This species is known only from the type locality and the character of the seed ornamentation could be of taxonomic importance to distinguish it from other related species (Ferren 2003). These investigations show that the easily visible detachment of the aril from the seed coat, appearing as a patch near the cotyledon area, clearly distinguishes *S. verrucosum* from other taxa encountered in Africa. This character is added to the diagnostic key as a taxonomically important trait. Apart from *S. verrucosum*, this peculiarity is also observed in the North American annual *S. maritimum* and South American *S. parviflorum* DC., a forgotten name of a species that is often identified as *S. portulacastrum* or *S. sessile* Pers. (Sukhorukov et al., in prep.). *S. verrucosum* and *S. maritimum* appear closely related to each other according to the molecular data (Bohley et al. 2015). Other American species previously considered within the genus *Cypselea* and recently transferred to *Sesuvium* based on the molecular phylogeny (Bohley et al. 2017) – *Sesuvium humifusum* (Turpin) Bohley & G.Kadereit, *S. mezianum* (K.Müll.) Bohley & G.Kadereit and *S. rubriflorum* (Urb.) Bohley & G.Kadereit – have much smaller, reddish seeds with a thin seed coat (Sukhorukov, pers. observ.). The seeds of these three species (~0.2 mm across) are amongst the smallest in the large “Globular Inclusion” clade (core Caryophyllales: Cuénoud et al. 2002) along with tiny seeds of some Molluginaceae (Sukhorukov et al., in press).

Many African taxa with an annual or perennial life history (*S. congense*, *S. crithmoides*, *S. crystallinum*, *S. digynum*, *S. sesuvioides*) possess an indistinctly striate seed surface (Figs 2 and 3; see also Sukhorukov et al. (2017)). Smooth seeds of *Sesuvium sesuvioides* or indistinctly wrinkled seeds of *S. digynum* have relatively thin (20–30 µm) testa. However, thickness varies considerably in *S. hydaspicum* (from 20 to 50 µm) and especially in *S. nyasicum* (from 20 to 100 µm) due to the presence of protruding “wrinkles” originating from the testa. The testa is thinner between the wrinkles and much thicker in wrinkled areas.

### Molecular phylogeny

**Figure 5.**
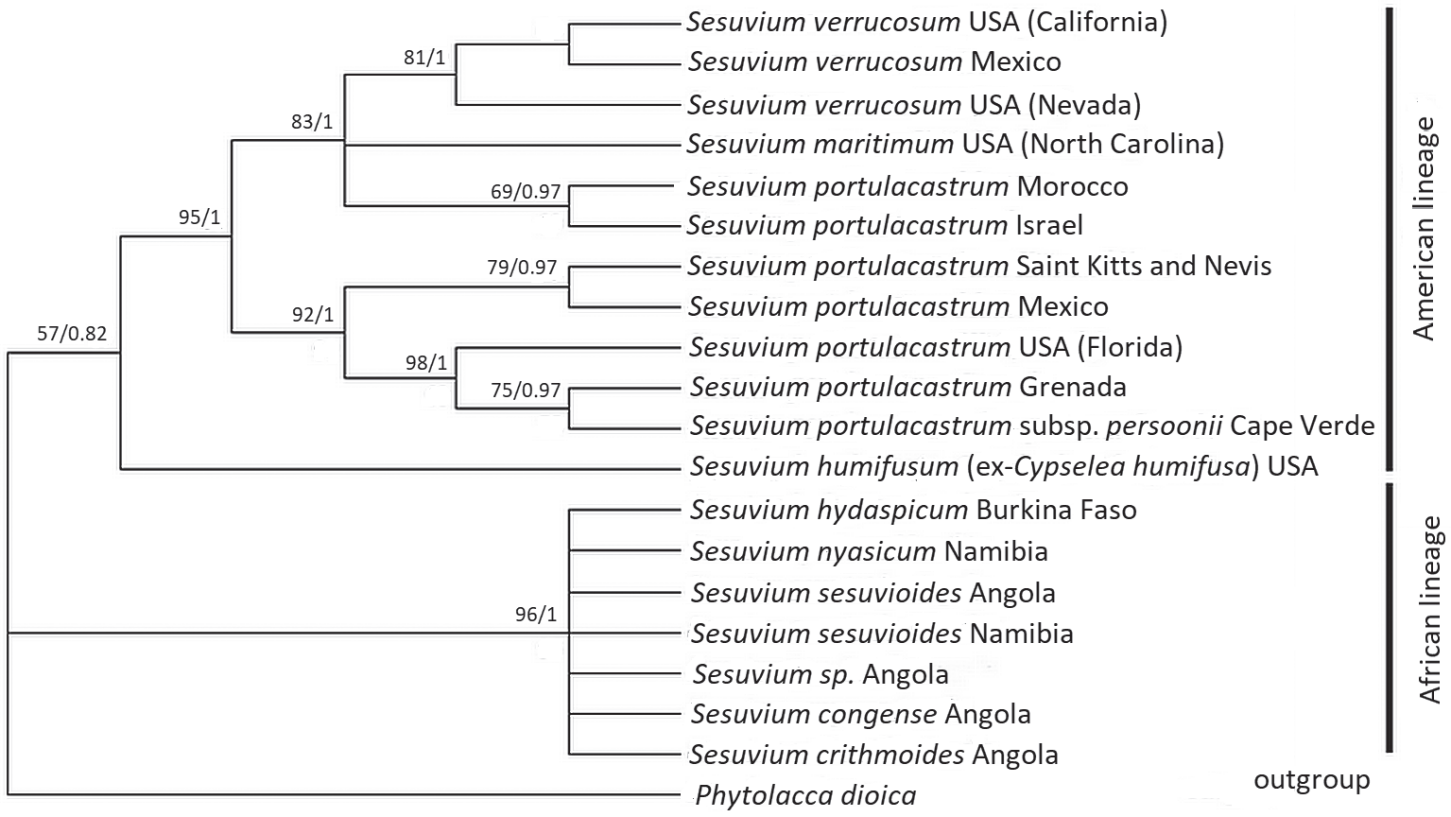
Phylogenetic relationships of perennial *Sesuvium* species from ML analysis of combined plastid sequences (rps 16 intron, trnL-trnF, atpB-rbcL, 1377 bp in total). The tree is drawn to scale, with branch lengths measured in the number of substitutions per site. ML bootstrap support/BI posterior probabilities are specified at the branch nodes (not shown when <50%).

**Figure 6.**
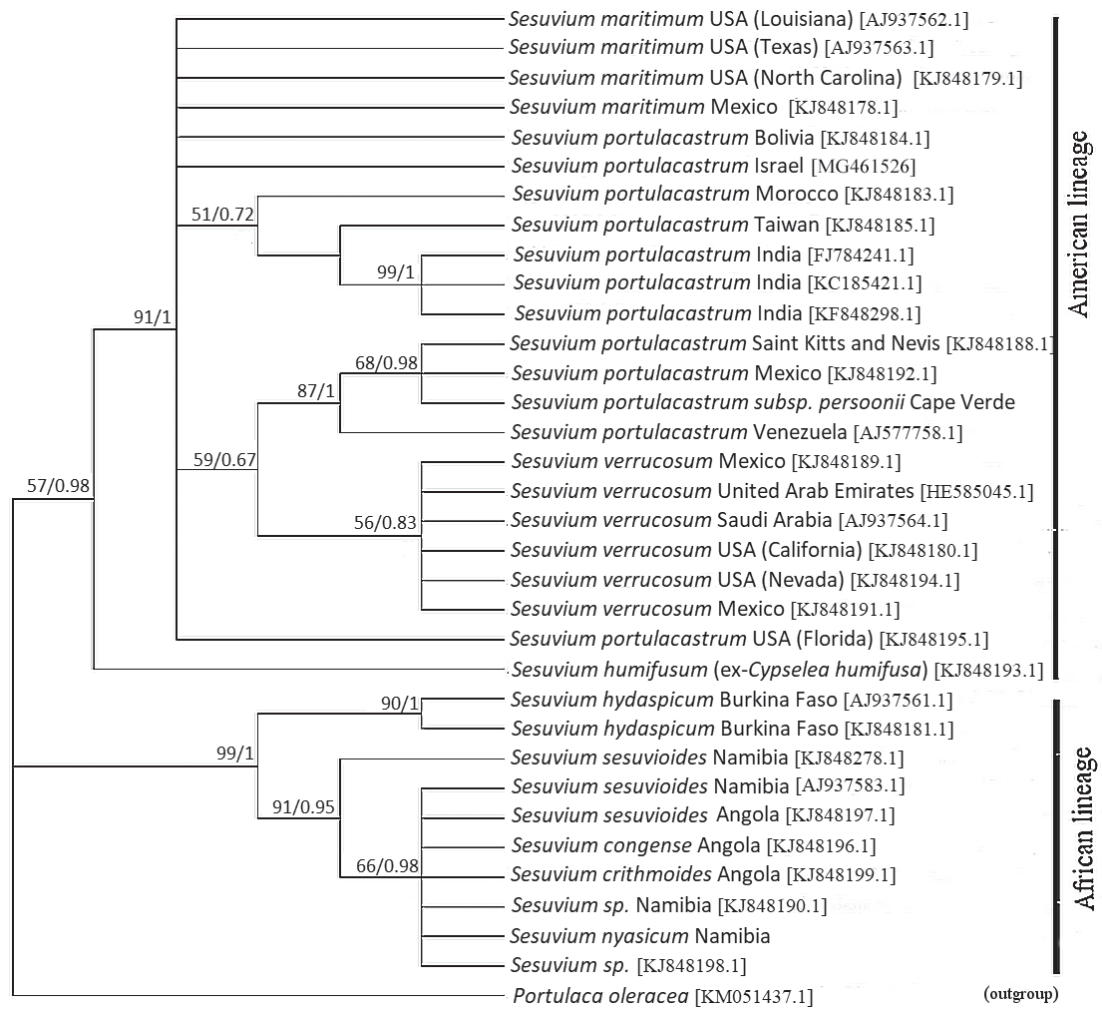
Phylogenetic relationships of perennial *Sesuvium* species from ML analysis of ITS sequences. The tree is drawn to scale, with branch lengths measured in the number of substitutions per site. ML bootstrap support/BI posterior probabilities are specified at the branch nodes (not shown when <50%).

**Figure 7.**
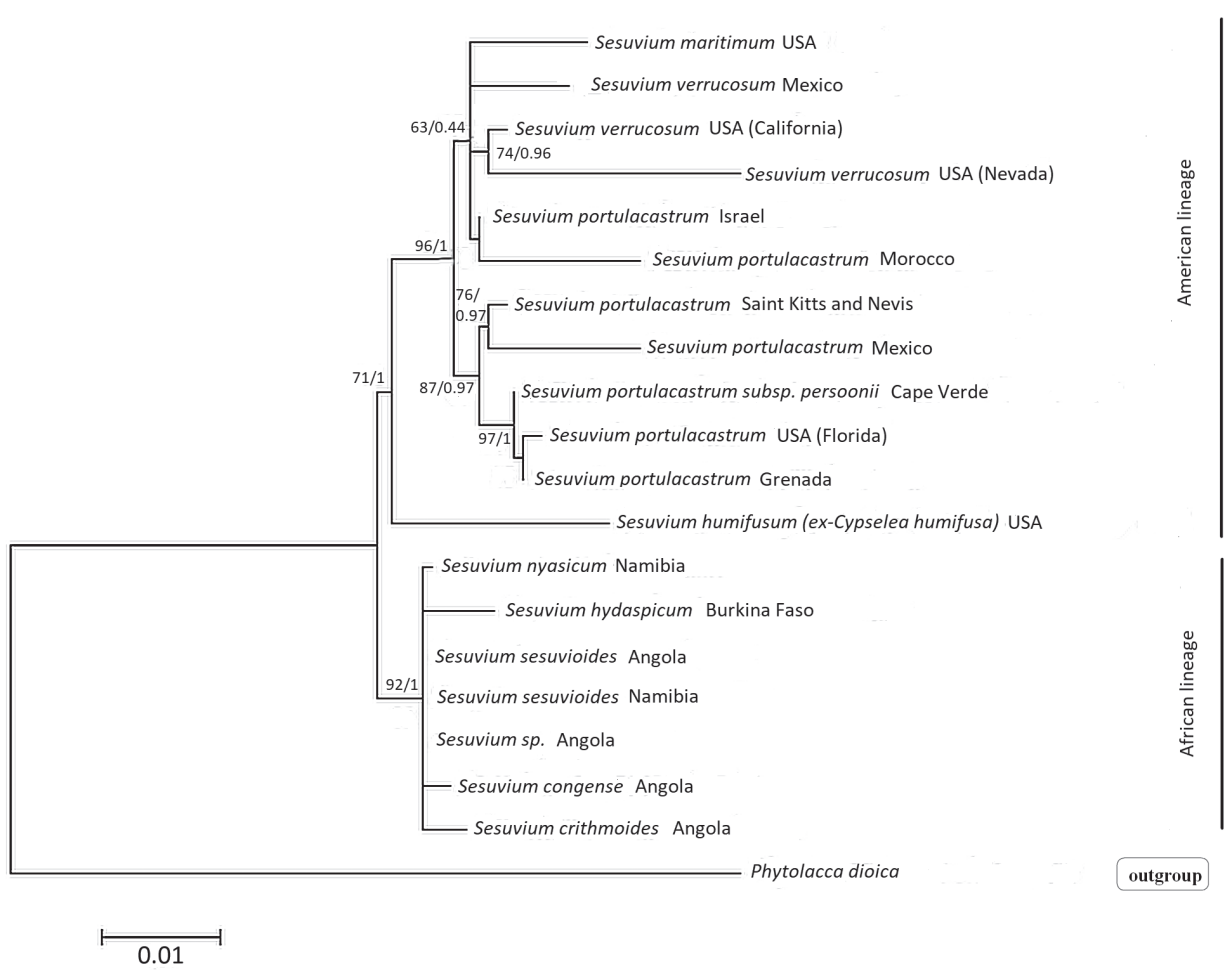
Phylogenetic relationships of perennial *Sesuvium* species inferred from combined analysis of plastid (rps 16 intron, trnL-trnF, atpB-rbcL) and ITS sequences. The tree is drawn to scale, with branch lengths measured in the number of substitutions per site. ML bootstrap support/BI posterior probabilities are specified at the branch nodes (not shown when <50%).

Several new samples were added to the molecular phylogeny including *S. nyasicum*, *S. portulacastrum* and the new subspecies S. portulacastrum subsp. persoonii. In both ITS and chloroplast trees (Figs 5 and 6), as well as in the combined tree (Fig. 7), *Sesuvium* is divided into two clades referred to as the “African” and the “American” lineages (Bohley et al. 2015). Although the relationships within the “African lineage” are still not resolved, this clade contains the species native to Africa. In contrast, the “American lineage” consists of the species originating in America, including samples of *S. portulacastrum* collected in Asia and Africa. In all trees, *Sesuvium portulacastrum* is not monophyletic. The African Sesuvium portulacastrum subsp. persoonii is nested within the “American lineage” as a part of the *Sesuvium portulacastrum* complex, either as a sister lineage to *S. portulacastrum* (the sample from Grenada) in the chloroplast tree (Fig. 5) or amongst the Central American samples of *Sesuvium portulacastrum* complex (Fig. 6). Due to its well-defined distribution range, this new taxon with clearly petiolate, shorter and thicker leaves is considered here as a subspecies of *S. portulacastrum*. However, the taxonomic status of S. portulacastrum subsp. persoonii needs further studies for the following reasons: (1) the lack of material from the Indian subcontinent, especially *S. repens* Willd. and *S. portulacastrum* (s.l.) from the Americas, Africa (e.g. *S. ayresii*) and Southeast Asia, precludes recognition of the exact relationships of all taxa within the large “American lineage” and (2) *Sesuvium portulacastrum* is still considered a highly variable species distributed worldwide (Bohley et al. 2017). However, some “strange” forms of this species in Asia (especially in the large biogeographical region of Malesia) are present in the European herbaria in a very limited quantity and were not included in the molecular analysis. The preliminary morphological studies (Sukhorukov et al., in prep.) suggest that at least two species need to be reinstated to species rank (*S. parviflorum* DC. and *S. microphyllum* Willd.) and some new taxa from South and Central America are yet to be described.

### Taxonomy of perennial *Sesuvium* in Africa

One American species (*S. verrucosum*) and one new subspecies (S. portulacastrum subsp. persoonii) are added to the taxonomic list of *Sesuvium* in Africa. The authors also propose to merge *S. crystallinum* with *S. crithmoides*. According to the latest investigations in Angola, *S. sesuvioides* previously considered as an annual species (e.g. Gonçalves 1970, Sukhorukov et al. 2017) can be a facultatively short-lived perennial herb. In total, six perennial species in Africa (*S. ayresii*, *S. congense*, *S. crithmoides*, *S. portulacastrum*, *S. verrucosum* and the facultatively short-lived *S. sesuvioides*) and one subspecies of *S. portulacastrum* mentioned above have been accepted.

### Diagnostic key to perennial *Sesuvium* in Africa

**Table:** 

1	Stems and leaves densely papillate (plants grayish); flowers sessile or with short (up to 3 mm) pedicels	**2**
–	Stems and leaves glabrous (younger leaves may be papillate); flower sessile or pedicellate (pedicels up to 15 mm)	**4**
2	Each flower surrounded by 4(–6) bracteoles	***S. crithmoides***
–	Each flower with 2 bracteoles	**3**
3	Old stems stout, hardened; leaves linear to lanceolate (lower leaves often spatulate); perianth cup (concrescent part of the segments) roundish; aril tightly adherent to the seed coat	***S. congense***
–	Old stems not hardened; leaves oblong; perianth cup turbinate; aril peeling off the seed coat near the cotyledon area (appearing as a white fold)	***S. verrucosum***
4	Leaves up to 25(28) mm long; flowers sessile or shortly pedicellate (pedicels up to 3.5 mm)	**5**
–	Leaves usually longer; pedicels 7–12(20) mm long	**6**
5	Perennial; leaves terete or semi-terete; flowers sessile or shortly pedicellate (pedicels up to 3.5 mm), white or pink	***S. ayresii***
–	Short-lived perennial or annual; leaves conduplicate; flowers sessile, mauve	***S. sesuvioides***
6	Ramification not rampant; leaves clearly petiolate (petioles 5–10 mm long), usually less than three times longer than wide (all blades including those of upper leaves ovoid or oblong, 20–40 × 10–15 mm), and very fleshy (3–9 mm thick)	**S. portulacastrum subsp. persoonii**
–	Ramification rampant; leaves shortly petiolate (petioles up to 3 mm long), more than three times longer than wide (all blades oblong-spatulate or oblanceolate, 20–60 × 5–10(12) mm) and thinner (1.5–4 mm)	**S. portulacastrum subsp. portulacastrum**

### Synopsis of perennial *Sesuvium* in Africa

#### Sesuvium ayresii

Taxon classificationPlantaeORDOFAMILIA

Marais, Kew. Bull. 32(2): 483 (1978)

Fig. 8

##### Holotype.

MAURITIUS [main island], Fort William, Sep 1860, *Ph.B. Ayres s.n*. (K000076290! iso – LE!).

##### Description.

The description of *S. ayresii* was provided by Marais (1978). The most indicative characters of this species are small (up to 25–28 mm long, but usually smaller) terete or semi-terete leaves and (sub)sessile flowers (see Marais 1978, Hartmann 2002). Additionally, Marais (1978) reported a smaller number of stamens (12–20) that have never been observed in *S. portulacastrum* (stamens more than 30). The smaller seed size (~1 mm) of *S. ayresii* compared with *S. portulacastrum* (Marais 1978) seems to be an insignificant diagnostic trait. Leaf shape and leaf size are very variable, sometimes within a given individual.

**Figure 8.**
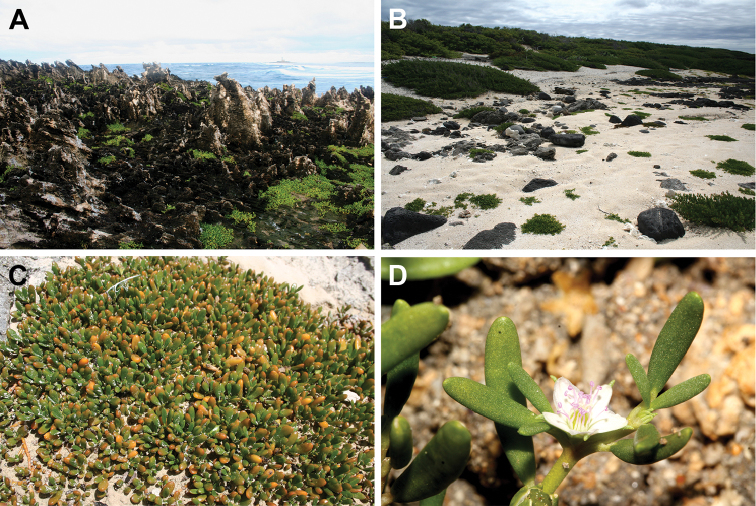
*Sesuvium ayresii*: **A** the only species growing on the islet (Ile aux Fous, Mauritius, 1 August 2007) **B** clumps on sandy beach (Ilot Gabriel, Mauritius, 6 August 2007) **C** an individual clump on calcarenite (Ile de la Passe, Mauritius, 3 February 2007) **D** close-up of a flower (Rivulet Terre Rouge Bird Sanctuary, Mauritius, 1 September 2017). Photographs by F.B.V. Florens.

##### Ecology.

*Sesuvium ayresii* usually grows on coral rocks, basalts or calcarenites (Marais 1978), but it also can be encountered on sandy seashores, like many other species of the genus. The records of *Sesuvium* from the calcarenite islets of Les Bénitiers (Johnston 1894) and Rochers des Oiseaux (Johnston 1895) probably belong to *S. ayresii*. *Sesuvium ayresii* is reported as the only member of the genus in the Mascarenes (Marais 1990).

##### Additional specimens examined

(Fig. 14). MAURITIUS [main] Island: Gris Gris, [no date, before 1932], *Vaughan 653* (MAU 0017795); Rocky coast near Rivière des Anguilles, 8 Dec 1962, *Edgerley S.n*. (MAU 0017801); Post Lafayette, east coast, 11 Jan 1973, *Lorence 189* (K, MO324309); estuary of Black River, 10 Sep 1981, *L. Averyanov 446* (MHA); Mer Rouge, 13 Mar 2004, *Pynee et al. s.n.* (MAU 0012461); Cap Malheureux, 26 Apr 2004, *Pynee S.n*. (MAU 0017803); Albion, 10 Nov 2011, *Pynee S.n*. (MAU 0009004); Rivulet Terre Rouge Bird Sanctuary, 01 Sep 2017, *Baider CB 2729 & V. Florens* (MAU 0023815); Mauritius [islets] Island: Gunner’s Quoin or Quoin de Mire, 1871, *Horne 129* (K); 06 Aug 2007, *Baider CB 677 & V. Florens* (MAU 0023819); 07 Aug 2007, *Baider CB 701A & V. Florens* (MAU 0023820); 07 Aug 2007, *Baider CB 701B & V. Florens* (MAU 0023821); Ile de la Passe, 26 Oct 1888, *Johnston s.n*. (E00651982); 29 Nov 2003, *Baider CB 588 & V. Florens* (MAU 0023826); Ile aux Fouquets, 4 Nov 1962, *Rountree s.n.* (MAU 0017798); Ilot Marianne, east coast, 13 May 1956, *Vaughan s.n.* (MAU 0017796); 18 Jan 1975, *D. Lorence 1059* (K, MAU 0017800); 28 Nov 2007, *Baider CB 551 & V. Florens* (MAU 0023822); 31 Jul 2007, *V. Florens s.n.* (MAU 0023823); Ilot Sancho, south coast, 15 Aug 1974, *D. Lorence 943* (K, MAU 0017799); Ile D’Ambre, 21 Dec 2003, *Baider CB 783A* & *V. Florens* (MAU 0023827); 21 Dec 2003, *Baider CB 783B* & *V. Florens* (MAU 0023828); Ilot Bernache, 21 Dec 2003, *Baider* CB 814 & *V. Florens* (MAU 0023829); Ilot Gabriel or Gabriel Islet, 20 Apr 2006, *Pynee S.n*. (MAU 0017804, MAU 0017805), 06 Aug 2007, *Baider CB 1942*, *V. Florens & D. Hammond* (MAU 0023825); Ile aux Fous, 01 Aug 2007, *V. Florens & D. Hammond s.n.* (MAU 0023824); Rodrigues [main] Island: Plaine Coral, Jul 1970, *Cadet RO218/2604* (MAU 0017807); 1874, *Balfour s.n*. (E00651981, K); Rodrigues [islets] Island: Frigate Island, Jan 1963, *Staub s.n.* (MAU 0017806); Ile Gombrani, 10 Jan 2004, *Baider CB 932 & V. Florens* (MAU 0023817); Ile aux Crabes, 13 Jan 2004, *Baider CB 1036 & V. Florens* (MAU 0023818); Ile aux Cocos, 15 Jan 2004, *V. Florens s.n.* (MAU 0023816).

##### General distribution.

Endemic to the Mascarenes.

##### Conservation status.

The species should be considered Near Threatened (NT) according to the IUCN red list criteria (IUCN 2017). This assessment is based on the species’ EOO of 24,241 km^2^ and AOO of 68 km^2^; together with other factors including the species’ habitat being restricted to seashores affected by salt spray, fragmentation of the populations and a high probability of losing sites in the near future due to habitat transformation (construction of hotels, improvement of seashores by removal of vegetation, dumping of refuse in the coastal belt), especially on mainland Mauritius. Only a few of the populations are located in areas with some degree of protection such as Nature Reserves or National Parks (one on Rodrigues; nine on Mauritius), most of them being on small islets. Some records are over 50 years old and need to be updated to determine any decline in its geographic distribution. Competition with invasive alien plants seems not to be a serious problem for this species, although sea-level rise is reducing the area of suitable habitat.

#### Sesuvium congense

Taxon classificationPlantaeORDOFAMILIA

Welw. in Oliver, Fl. Trop. Afr. 2: 586 (1871)

##### Lectotype

(Gonçalves 1965): [ANGOLA, Bengo Province] Dist. Ambriz, Habit. freq.[ent] in rupestribus et glareosis ad ostia flum. Onço in Mossul [Ambriz Municipality, frequent in mountainous and gravelly places along the estuary of the river Onço in Mosul] fl. & fr. Nov 1853, *Welwitsch 2382* (LISU214650 – photo! isolectotypes – BM000839899!, BM001209754! K000076293! LE!, P04602200!)

##### Nomenclatural notes.

A specimen in LISU has been wrongly stated to be the holotype by Gonçalves (1965) and then by Bohley et al. (2017). Indeed, the sheets of *S. congense* with the same label and collection number are present in several herbaria, as are many other specimens of Welwitsch’s material from Angola (Albuquerque et al. 2009). No specimens and herbarium were cited in the protologue (Welwitsch in Oliver 1871) except the location “Lower Guinea, Congo [Angola as a part of Kongo Kingdom], Ambriz”. The lectotype selected here is in accordance with Art. 9.9 of ICN (McNeill et al. 2012). The synonymisation of *S. congense* with *S. portulacastrum* (Adamson 1962) is incorrect.

The epithet “congense” probably refers to the “Kingdom of Kongo”, a West African kingdom that united the territories of northern Angola (incl. Bengo and Zaire provinces) and the western part of DR Congo, as well as portions of Republic of Congo and Gabon.

##### Description.

The morphological description of the species is provided in Oliver (1871), Gonçalves (1970) and Bohley et al. (2017). This species is sometimes confused with branched *S. sesuvioides* (especially when the upper parts of the branches are collected) with similar smooth seeds. In contrast to *S. congense* or related *S. crithmoides*, *S. sesuvioides* is glabrous, with turbinate or balustriform flowers (without a rounded perianth cup).

##### Additional specimens examined.

ANGOLA: **Benguela prov.**: Lengue, 19 Dec 1932, *Grossweiler 9715* (BM); 20 km W of Benguela, Baia Azul, 1 Apr 1973, *P. Bamps & S. Martins* 4372 (BR0000013827366); 74 km S of Benguela along road to Cuio, 74 m alt., 25 Dec 2016, *C. Klak 2557* (BOL); **Namibe prov**.: Maiombo river, Oct 1859, *Welwitsch 2395* (BM); Mossamedes [Namibe], valley of Rio Mukungo, Aug 1937, *H. Humbert 16407* (BM); Mossamedes [Namibe], Porto Alexandre, 26 May 1937, *A.W. Exell & F.A. Mendonça 2294* (BM); Mossamedes [Namibe], Porto Alexandre, Aug 1937, *H. Humbert 16375* (BM); ca. 22 km NE of Namibe, 18 Jan 2009, *Winter 7683* (PRE); road to Baba from Lucira road, 23 Jan 2009, *Winter 7779* (PRE); Namibe, 9.7 km S of airport turn-off, 23 Jan 2009, *Winter 7762* & *7766* (PRE); 27 km E of Namibe, 252 m, 19 Dec 2016, *C. Klak 2554* (BOL).

##### General distribution

**Figure 9.**
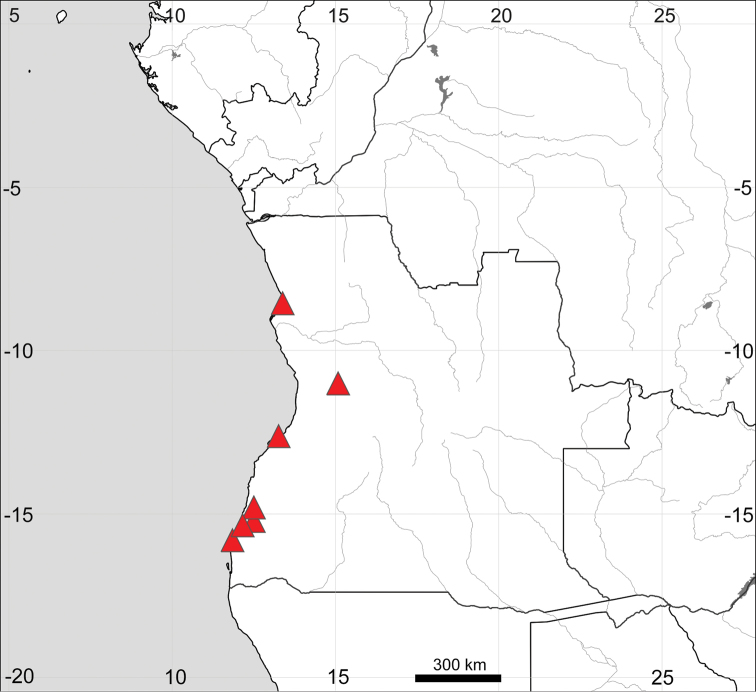
Distribution map of *Sesuvium congense*.

(Fig. 9). Coastal sandy areas in Angola, from Bengo to Namibe provinces, recorded at altitudes between 74 and 252 m a.s.l. (Gonçalves 1965).

##### Conservation status.

*Sesuvium congense* has an estimated EOO of 54,340 km^2^ (which would place the species in LC) and AOO of 36 km^2^ (which would place it in EN). However, it is unknown if the species persists in some of these localities. The size of its populations and their threats are little known, but the populations on the seashore and near rivers are probably impacted by development and agriculture. Therefore, the species, at this point in time, should be considered Data Deficient (DD) according to the IUCN Red List Criteria (IUCN 2017).

#### Sesuvium crithmoides

Taxon classificationPlantaeORDOFAMILIA

Welw., Ann. Conselho Ultramar. ser. 1: 586 (1859)

Figs 10 , 11

##### Lectotype

(designated here by Sukhorukov). ANGOLA, distr. Loanda [Luanda], in arenosis maritimis de Ilha de Loanda [on sandy seashores of Loanda Island], 12 Jun 1858, *Welwitsch 2386* (BM000839897! specimen on the left; isolectotypes – BM001209752! BM001209753! K000076292! P04602195! COI00070549! [photo seen], LISU031837! [photo seen]).

**Figure 10.**
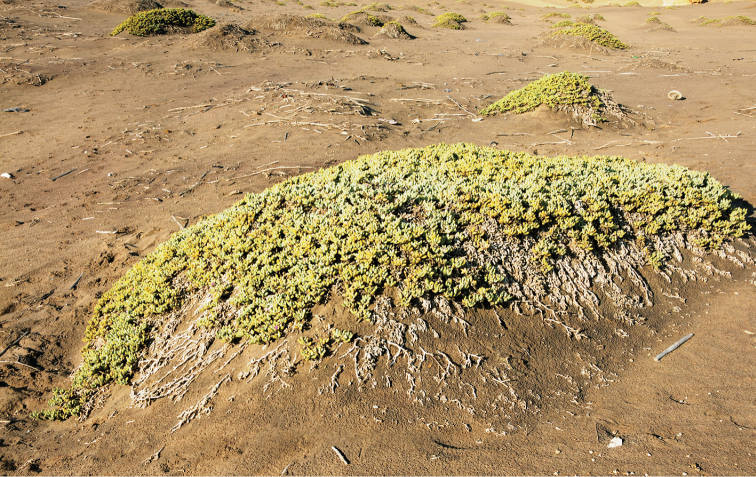
General view of *Sesuvium crithmoides* (incl. *S. crystallinum*) on the dunes of Rio dos Flamingos, Angola. Photographs by C. Klak and P.V. Bruyns (December 2016).

**Figure 11.**
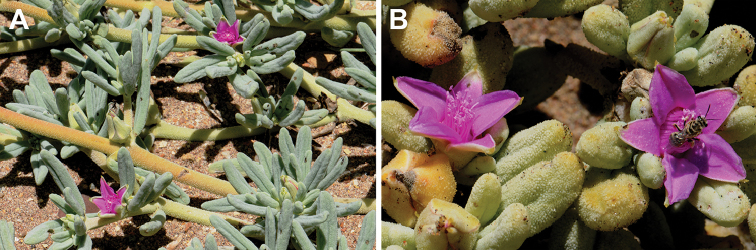
Parts of the plant of *Sesuvium crithmoides*: **A** reproductive shoots **B** close-up view of flowers. Photographs by C. Klak and P.V. Bruyns (at the mouth of Rio dos Flamingos, south of Namibe, Angola, December 2016).

##### Note.

Welwitsch collected this new species in 1854 and 1858 from several neighbouring locations in Luanda Province. All examined sheets were labelled with the same collector’s number (2386) and the location of the lectotype specimen is close to that mentioned in the protologue (Barra do Dande settlement, ca. 30 km N of Luanda). Surprisingly, none of the authentic specimens contained the name of Barra do Dande (Welwitsch 1859) and the species itself was not mentioned in a subsequent treatment of the genus (Welwitsch in Oliver 1871).

##### –Sesuvium mesembryanthemoides Welw., Ann. Conselho Ultramar. ser. 1: 557 (1859), nomen nudum

**Note.** Welwitsch (1859) mentioned the name *Sesuvium mesembryanthemoides* (nomen nudum) for the first time, but did not describe the plant morphologically (“Uma nítida espécie de *Sesuvium*” [a distinct species of *Sesuvium*]). He probably supposed that it was conspecific with *S. crithmoides*, which was described in the same article (Welwitsch 1859). As mentioned above, all sheets of *S. crithmoides* and S. mesembryanthemoides (nomen), collected by Welwitsch, have the same collection number (2386).

##### =*Sesuvium mesembryanthemoides* Wawra in Wawra & Peyr., Sitzungsber. Acad. Wien, Math.-Nat. 38: 564 (1860).

**Lectotype** (designated here by Sukhorukov). [ANGOLA] Benguela, *Dr Wawra 210* (LE!).

**Note.** Interestingly, Wawra collected the same species in Angola independently from Welwitsch and used the same epithet “*mesembryanthemoides*” for his new *Sesuvium*. Unfortunately, the original sheets of *S. mesembryanthemoides* Wawra cited in the protologue (“in littore maris prope Benguelam, *Wawra 210*”: Wawra and Peyritsch 1860) were destroyed in B, W or WU (Bohley et al. 2017; Johannes Walter, pers. comm.). Wawra and Peyritsch (1860) reported the presence of four to six bracteoles in the flowers of *S. mesembryanthemoides* and it therefore evidently differs from *S. congense* (with similar narrow leaves), which has flowers with two bracteoles only. Bohley et al. (2017) have designated the lectotype of *S. mesembryanthemoides* Wawra in the herbarium LISU (with isolectotypes in BM, BR, C, COI, K, LE) based on Welwitsch’s specimens (“Mossamedes [Namibe], seashore, 1 Jul 1859, *Welwitsch 2389*”). However, the material collected by Welwitsch in Namibe province of Angola is not mentioned in the protologue of *S. mesembryanthemoides* Wawra and does not belong to the original material. Therefore, this lectotypification cannot be accepted. A lectotype using a Wawra’s specimen (syntype) seen in LE has been selected.

##### =*Sesuvium crystallinum* Welw. in Oliver, Fl. Trop. Afr. 2: 586 (1871).

**Lectotype** (designated here by Sukhorukov): [ANGOLA] Mossamedes [Namibe], hab.[itat] in arenosis maritimis pr.[ope] Mossamedes [on sandy seashores near Mossamedes], Jul 1859, *Welwitsch 2389* (BM000839898! isolectotypes – C, COI, G! K! LE! LISU).

Two locations (“Mossamedes” and “Benguela”) were indicated in the protologue. The lectotype of *Sesuvium crystallinum* is selected here from the specimens collected by Welwitsch with the number *2389* which were located in different herbaria including LISU (“holotype” in Bohley et al. (2017); not correctable to “lectotype” under Art. 7.10).

**Taxonomic and nomenclatural notes.** The type material of *S. crithmoides* comprises the plant fragments with narrow (linear or lanceolate) leaves reaching 8 cm in length. The leaf length and shape is a single character used for its delimitation from the closely related *S. crystallinum* (Gonçalves 1970) and *S. mesembryanthemoides* Wawra (Bohley et al. 2017). Both species are considered to have shorter (up to 5 cm) and broader leaves. However, the authentic material and protologue of *S. mesembryanthemoides* clearly state that this plant was described as a remarkable species with subtriquetrous-terete (narrow) leaves (Wawra in Wawra and Peyritsch 1860). Therefore, the use of *S. mesembryanthemoides* as a priority name against *S. crystallinum* (Hartmann 2002, Figueiredo and Smith 2008, Bohley et al. 2017) with broader and shorter leaves cannot be accepted. In all characters, including leaf length and shape, *S. crithmoides* and *S. mesembryanthemoides* are clearly conspecific.

The authors propose to merge the broad-leaved individuals (*S. crystallinum*) with *S. crithmoides* for the first time. Observations by the authors in Angola (C. Klak and P. Bruyns) did not confirm the separate existence of “short-leaved” or “long-leaved” plants. Other morphological and carpological characters are the same in both *S. crithmoides* and *S. crystallinum*. Only *S. crithmoides* (*Winter 7786* (PRE) from Baba, Angola) was included in the molecular analysis (Bohley et al. 2017).

*Sesuvium crithmoides* was considered as an endemic to Angola, although with possible records in coastal areas of the DR Congo (Bohley et al. 2017). One collection of *S. crithmoides* from the DR Congo (see also Hauman 1951, sub *S. mesembryanthemoides*) has been found and was also identified for the Republic of Congo for the first time (previously wrongly labelled as *Sesuvium portulacastrum*). All specimens seen from the Republic of Congo or the DR Congo have long and narrow leaves.

##### Additional specimens examined.

ANGOLA: **Benguela prov.**: Benguela, [without date] *H. Vanderyst 13141* (BR0000013827410); near Benguela, Lobito Bay, 1 Sep 1906, *H. Bolus 12453* (BOL); S of Benguela, seashore at Cuio village, 25 Dec 2016, *C. Klak 2558* (BOL); **Cabinda prov**: Landana, 9 Aug 1895, *A. Dewevre 231* (BR0000013827380), Landana, 15 Aug 1913, *Bequaert 616* (BR000000871151); Cabinda, Sumba village, 30 Nov 1957, *Lebrun 11195* (BR0000013827441; K); **Cuanza Sul prov.**: Praia de Sousa, 11°36'S 13°47'E, 3 Feb 1975, *J.D. Ward 82* (K, WIND); **Luanda prov.**: Luanda, *Welwitsch 2380* (LE), the same place, 13 Sep 1955, *J. Lebrun 10905* (BR0000013827403); **Namibe prov.**: Cabo Negro, Sep 1859, *Welwitsch 2387* (BM); Cabo Negro, Aug 1937, *H. Humbert 16391* (BM); the same place, 15 Apr 1973, *P. Bamps et al. 4519* (BR0000013827465); Mossamedes [Namibe city], 1937, *L.W. Carrisso and F. Sousa 218* (BM); Mossamedes, 21 Sep 1955, *J. Lebrun 10926* (BR0000013827472); Baba, 23 Jan 2009, *P.J.D. Winter 7786* (LUBA, PRE); seashore at mouth of Rio dos Flamingos, 17 Dec 2016, *C. Klak 2551* (BOL); DEMOCRATIC REPUBLIC OF CONGO: **Kongo Central prov**.: Banana, [without date] *Gillet S.n*. (BR0000013827434); [Nature Reserve] Luki-Mayumbe, 1959, *Flamigni 10773* (BR0000013827427); REPUBLIC OF CONGO (new records): Kouilou, 5 Sep 1962, *L. Makany 63* (P04602222); Djeno Region [Pointe-Noire], 26 Jan 1966, *C. Farron 4795* (P04602197 & P04602199); Pointe-Noire, Dec 1958, *J. Koechlin 5528* (P04602193).

##### General distribution

**Figure 12.**
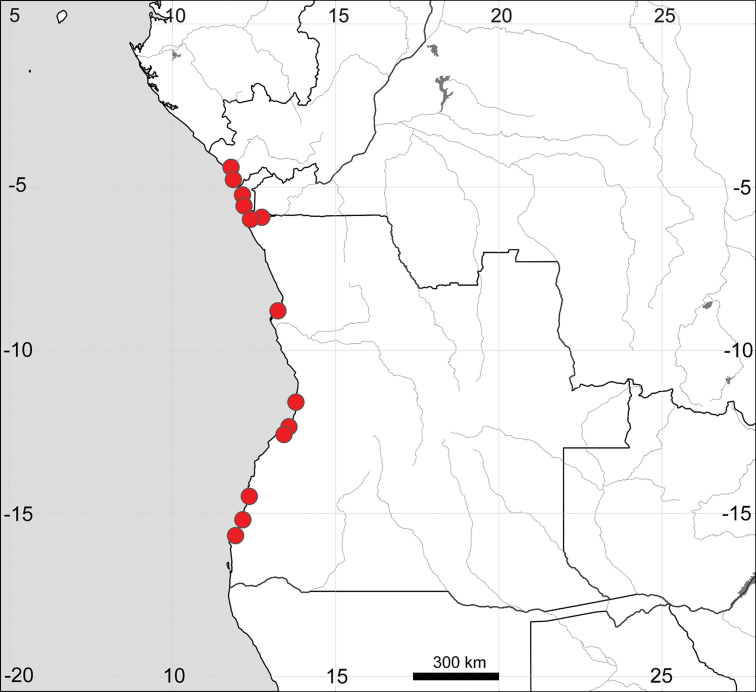
Distribution map of *Sesuvium crithmoides*.

(Fig. 12). Angola, Democratic Republic of Congo, Republic of Congo. *Sesuvium crithmoides* has been introduced to USA (Georgia, Glynn county, Brunswick, on ballast, 15 Aug 1902, *R.M. Harper 1524* (BM!); see also Small (1933)), probably as casual and not naturalised species (Ferren 2003). The specimen seen also has long and narrow leaves.

##### Conservation status.

*Sesuvium crithmoides* has an estimated EOO of 177,271 km^2^ and AOO of 56 km^2^. It was found to be common in two localities in Angola (*C. Klak 2551* & *2558*), where it grows within 50 metres of the sea. Sources of disturbance include vehicles driven along the beach, which was observed near Namibe city. However, vehicles are even now rather few in Angola and much of the southern, very arid Angolan coastline is still relatively pristine. Due to its large EOO and low threat level, the authors therefore recommend this species to be classified as Least Concern (LC) according to the IUCN Red List Criteria (IUCN 2017).

#### Sesuvium portulacastrum

Taxon classificationPlantaeORDOFAMILIA

(L.) L., Syst. Nat., ed. 10(2): 1058 (1759).

≡Portulaca portulacastrum L., Sp. Pl. 1: 446 (1753).

##### Lectotype

(Wijnands 1983). Hermann (1698), Icon. 212 [112, a typographic error], as “Portulaca corassavica …”.

Two subspecies of *S. portulacastrum* growing in Africa have been accepted.

#### Sesuvium portulacastrum subsp. portulacastrum

Taxon classificationPlantaeORDOFAMILIA

Fig. 13

portulacastrum =Sesuvium brevifolium Schumach. & Thonn. in Schumacher, Beskr. Guin. Pl.: 233 (1827).

##### Lectotype

(designated here by Sukhorukov): Danish Gold Coast, Guinea [probably SE Ghana], *P.E. Isert s.n*. (C10004542! [photo seen]).

The lectotype is chosen due to inclusion of two elements in the protologue (Schumacher 1827), a specimen cited and a drawing (Table 216, Fig. 1).

**Figure 13.**
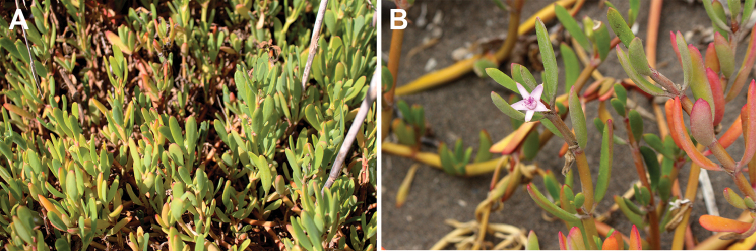
Parts of the plant of Sesuvium portulacastrum subsp. portulacastrum: **A** vegetative shoots **B** reproductive shoots. Photographs by M. Salas-Pascual (Gran Canaria, Canary Islands, Spain, July 2017).

##### Taxonomic notes.

The autonymous subspecies is of American origin and is known in many parts of tropical Africa and other continents, especially in regions with a hot and humid climate. According to the lectotypification undertaken by Wijnands (1983), the “true” *S. portulacastrum* is present in Central America (including the Caribbean Islands). The following characters distinguish this subspecies: rampant ramification, glabrous stems and adult leaves with mamillate epidermis, petioles up to 3 mm, oblong-spatulate leaves of 20–60 × 5–10(12) mm and 1.5–4 mm thick, conspicuous (7–12 mm) pedicels, flowers 10–15 mm in diameter and slightly elongated seeds. This description makes sense, because the species is non-uniform in its morphological characters (e.g. leaf length, presence of papillae on stems and leaves, seed ornamentation) and is corroborated by the molecular phylogeny (Bohley et al. 2017). Although *S. portulacastrum* is considered to have numerous synonyms (Bohley et al. 2017), at least some of them need further studies due to the presence of morphological differences, e.g. *S. microphyllum* Willd. (Caribbean Islands), *S. sessile* Pers. (South America?) or populations growing in Southeast Asia. In addition, *Sesuvium* is represented in Central America by at least six taxa (Sukhorukov et al., in prep.) and two of them have to be described as new species.

From humid coastal parts of West Africa, only one perennial species was described, *S. brevifolium* Schumach. & Thonn. (Schumacher 1827). This species has spatulate or oblong leaves with very short petioles, the characters being typical of *Sesuvium portulacastrum*. For this reason, *S. brevifolium* is merged with S. portulacastrum subsp. portulacastrum, this being in agreement with other accounts (Hooker 1849, Welwitsch in Oliver 1871, Bohley et al. 2017).

The autonymous subspecies of *S. portulacastrum* is distributed along the sea shores of many parts of tropical and subtropical Africa (Exell 1944, Jeffrey 1961, Gonçalves 1979, Gilbert 1993, Friedmann 1994, Sosef et al. 2006, Lisowski 2009, Acebes-Ginovés et al. 2010) and it seems to be present in almost all regions of Africa except South Africa. The causes of such invasion to seashore communities in Africa or in other regions of the Old World are not clear. It can be partially explained by the cultivation of *S. portulacastrum* in some areas for ornamental purposes, but mostly by transportation of its seeds in the sand ballast of ships sailing between America and other parts of the world in the 15^th^–17^th^ centuries. The examination of the herbarium specimens indicates that *S. portulacastrum* was sometimes collected in the same places as native *Sesuvium* (*S. congense* or *S. crithmoides*), e.g. on seashores of Kongo-Central province (DR Congo) and Angola.

##### Additional specimens examined.

ANGOLA: Luanda, Praia do Bispo, Dec 1858, *Welwitsch 2385* (BM); [Bengo prov.] Ambriz, [no date] *Welwitsch 2383* (K); [Bengo prov.] Dande River, 17 Sep 1955, *J. Lebrun 10908* (BR0000013828103); Mossamedes [Namibe], 10 Jan 1956, *E.J. Mendes* 1250 (BM); [Namibe prov.] Cabo Negro, 15 Apr 1973, *P. Bamps et al. 4522* (BR0000013828097, K, LE); Kwanza Sul prov., 10°51'S 13°48'E, 2 Feb 1975, *C.J. Ward and J.D. Ward 68* (K); BENIN: Cotonou beach, 22 Mar 1970, *L.A. Assi 11134* (G); DEMOCRATIC REPUBLIC OF CONGO: [Kongo Central prov.] Banana, 16 Jul 1915, *Bequaert 8014* (BR0000013828165); Bula-Bemba, 2 Sep 1958, *J. Wagemans 1982* (BR0000013828172); GABON: Estuaire prov., 22 Feb 1985, *A.M. Louis 1728* (BR0000013828028); GHANA: Sekondi, 3 Oct 1925, *H. Howes 980* (K); nr Tema harbor, 20 Sep 1960, *J.O. Ankrah 20547* (K); Accra, 12 Aug 1958, *J. Lebrun 11334* (BR0000013828042); Greater Accra Region, Ambassador Beach, 26 Feb 1977, *A.J.M. Leeuwenberg 11123* (BR0000013828035); GUINEA: Conakry, Aug 1954, *H. Jacques 7002* (LE); [Boké Region] Boffa pref., Bel-Air, 5 Feb 1979, *S. Lisowski 51828* (BR0000013827567); GLORIOSO ISLANDS: Iles aux Crabes (*C. Fontaine*, obs.; image seen!); KENYA: Kilifi distr., Malindi, 3 Dec 1961, *R. Polhill and S. Paulo 895* (BR0000013828059, K, P04602215); Mikindani distr., Mtwara, 12 Mar 1963, *H.M. Richards 17861* (K); Mombasa, 13 Dec 1969, *Bally 13736* (G); Tana River distr., Tana delta, Shekiko Camp, 25 Apr 1990, *S.A. Robertson 6121* (K); MADAGASCAR: [no exact location and date] herb. *Petit-Thouars s.n.* (P04600013); MOROCCO: Skhirat, 10 Jun 1937, *J. Gattefosse 138* (G, P05196618); MOZAMBIQUE (selected specimens): Delagoa [Maputo] Bay, 1890, *H. Junod 258* (G); Komati river, 15 Jul 1922, *C.E. Moss 7040* (BM); Lorenço Marques, 31 Aug 1959, *R. Watmaugh 313* (M); Maputo, 3 Jun 1970, *M.F. Correla and A. Marques 1630* (E00651988); Sofala province, Beira, 26 Feb 1972, *M.F. Correla and A. Marques 2812* (M); Maputo, 8 Mar 1979, *P.A. Schäfer 6707* (K); Inhambane prov., Massinga, Pomene, 20 Jun 1980, *J. de Koning 8197* (WAG1408388); Maganja da Costa, Praia Maraga, 15 Nov 1996, *A.R. Torre and M.F. Correia 14693* (BR0000013828134, M); [Massinga distr.] Pomene, 24 Sep 1980, *P.C.M. Jansen 7521* (BR0000013828110); SÃO TOMÉ & PRÍNCIPE: São Tomé [Island], Apr 1916, *A. Cortesão s.n*. (BM); SENEGAL: [Oussouye Dept.] Basse Casamance National Park, Kabrousse, 22 Dec 1976, *C. Van den Berghen 1582* (BR0000013827519); [Cap Vert Peninsula] Lake Retba, 20 Dec 1984, *D. Thoen 7367* (BR0000013827526); SEYCHELLES: Aldabra Island, 26 Feb 1968, *F.R. Fosberg 49547* (L1693568); Aldabra, South Island, Grand Cavalier, 11 May 1972, *D. Wood 1686* (E00651983); Farquhar Group, Farquhar Island, 2 Feb 1972, *Frazier 121* (K); Farquhar Group, St Pierre Island, 4 Oct 1941, *P.O. Wiehe 1681* (MAU 0023813, MAU 0023814); SIERRA-LEONE: Samu chiefdom, 22 Mar 1930, *R.R. Glanville 251* (BM, K); SOMALIA: Kodei village, 1°1'S 41°58'E, 29 Jun 1983, *J.B. Gillett et al*. *5116* (K); SPAIN: Canary Islands (selected specimens): Lanzarote, Playa Honda, 24 Mar 2011, *F. Verloove 9276* (BR); La Laja beach, Las Palmas de Gran Canaria, 28°03'38.70"N, 15°25'12.28"W, 31 Jul 2017, *M. Salas-Pascual s.n*. (MW); Beach of El Águila, San Bartolomé de Tirajana, 27°46°38.80"N, 15°31'38.50"W, 31 Jul 2017, *M. Salas-Pascual s.n*. (MW); El Veril beach, San Bartolomé de Tirajana, 27°45'36.78"N, 15°33'50.77"W, 31 Jul 2017, *M. Salas-Pascual s.n*. (BR, MW); Edge of the Charca de Maspalomas, San Bartolomé de Tirajana, 27°44'24.96"N, 15°35'43.79"W, 31 Jul 2017, *M. Salas-Pascual s.n*. (MW); TANZANIA: Tanga, Tanga Bay, 4 Nov 1929, *Greenway 1853* (K); Zanzibar, Marahubi Beach, 22 Apr 1961, *H. Faulkner 2814* (BR0000013828073); Dar es Salam, 26 Aug 1968, *M. Batty 284* (K); TUNISIA: pers. comm. R. El Mokni (photo!).

##### General distribution.

**Figure 14.**
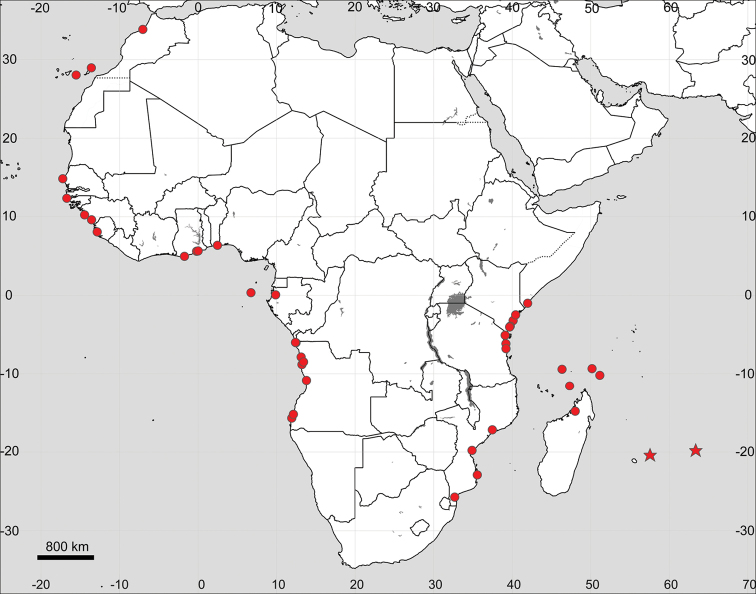
Distribution map of *Sesuvium ayresii* (stars) and S. portulacastrum subsp. portulacastrum (circles, mapped only for Africa).

The subspecies seems to be widely distributed on the seashores of the tropics, but some populations from tropical America and SE Asia are distinct in their morphological characters. The distribution of Sesuvium portulacastrum subsp. portulacastrum in Africa is presented in Fig. 14.

#### Sesuvium portulacastrum subsp. persoonii

Taxon classificationPlantaeORDOFAMILIA

Sukhor. subsp. nov.

urn:lsid:ipni.org:names:77174974-1

Sesuvium pedunculatum sensu Sieber (in herb.) non Pers.

##### Diagnosis.

Differs from the autonymous subspecies by the absence of rampant ramification, clearly petiolate leaves (petioles 5–10 mm long) that are usually less than three times as long as wide (all blades including those of upper leaves ovoid or oblong, 20–40 × 10–15 mm) and 3–9 mm thick.

##### Holotype.

Republic of Cape Verde, Sal Island, 2 km W of Santa Maria town, 16.590246, -22.924272, sandy depressions near the sea, 30 Aug 2015, *A.P. Sukhorukov 59* (MW0595660! iso – BR, G, K).

##### Description.

**Figure 15.**
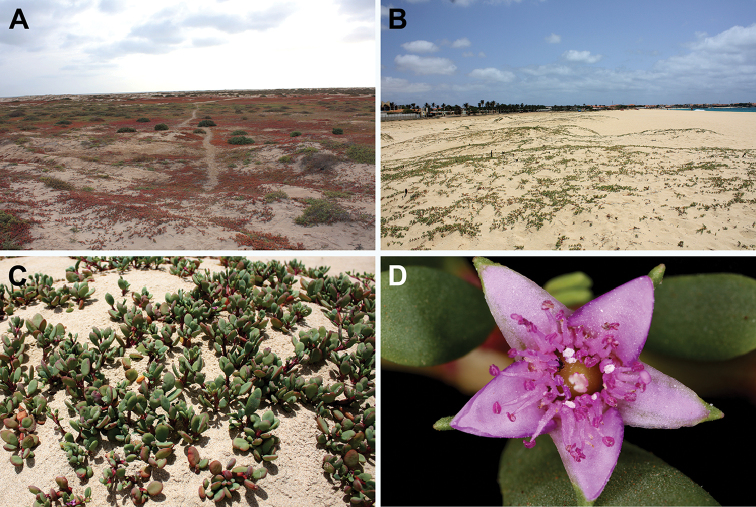
Sesuvium portulacastrum subsp. persoonii: **A** general view of the plant (of red colour) in saline depressions near the seashore, together with the subshrub *Arthrocaulon franzii* **B** S. portulacastrum subsp. persoonii on the seashore dunes **C** closer look at an individual **D** close-up of the flower. Photographs by A. Sukhorukov (**A–C** Sal Island, Cape Verde, August 2015) and A. Konstantinova (**D** Sal Island, Cape Verde, January 2016).

Sprawling glabrous perennial herb (the shoots are often partially buried by sand and appear to be separate plants) with ramification not rampant; stems rooted or not, roundish, greenish or more often red (Fig. 15A, B), 3–5 mm in diameter, ascendent (not creeping); leaves opposite, petiolate; petioles 5–10 mm, reddish or green, broadened basally, leaf blades oblong, 20–40 mm long (the leaves on the shortened shoots are smaller), 10–15 mm wide, 3–9 mm thick, entire, green or reddish (Fig. 15C); flowers solitary in the leaf axils (each node bears one flower from one of the opposite leaves), ~10 mm in diameter, with two hyaline glabrous bracteoles; pedicels 3–5 mm, accrescent at fruiting stage up to 10(15–20) mm long; perianth bifid, apically acutish, green abaxially and pink adaxially (Fig. 15D), without prominent red glands at the tip of the segments; stamens ~50, pink, slightly shorter than perianth, filaments 5 mm long, anthers 0.4–0.6 mm long; ovary turbinate, with (2)3–4 stigmas; seeds ~20, black, roundish, ~1 mm across, completely covered with a funicular aril; seed surface smooth or slightly uneven.

##### Etymology.

The subspecies is named after Christiaan Hendrik Persoon (1761–1836), botanist and mycologist, who described several *Sesuvium* species.

##### Ecology.

Sandy beaches near the sea and seasonally flooded, saline plains on the landward side of the coastal dune belt.

##### Flowering and fruiting.

All year round, but most abundantly from September to May (at least in the Cape Verde Islands).

##### Taxonomic and nomenclatural notes.

Franz Wilhelm Sieber labelled his *Sesuvium* collections from Senegal as *S. pedunculatum* Pers. The use of this name for the African material is very confusing but explained here.

The name was published by Persoon (1806), who provided a very short diagnosis mentioning pedicellate flowers (not petiolate leaves!) and noted that the species originates from India. It is assumed that Persoon probably did not see the plant in the wild. A specimen was found in the De Candolle herbarium (G-DC) that contains three fragments of different origin: two fragments of *S. portulacastrum* from the Caribbean and one fragment of Sieber’s collection from Senegal (1825) named *S. pedunculatum*. However, the material kept at G-DC is not a type of *S. pedunculatum*, but only one of the duplicates sent by Sieber to different herbaria.

**Figure 16.**
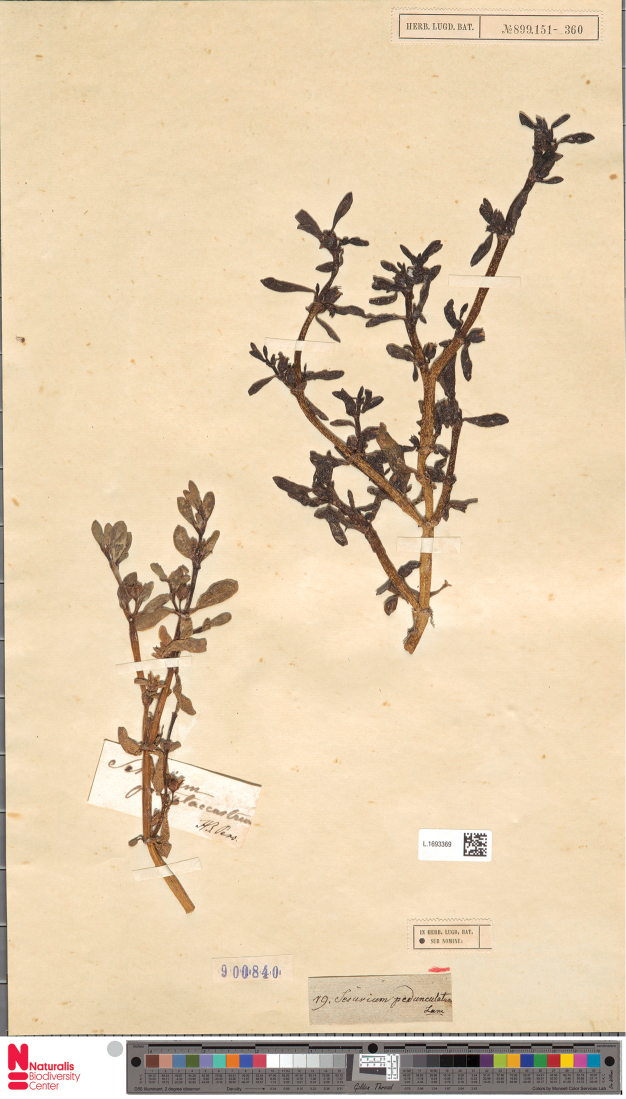
A specimen kept in Leiden (L1693369) and probably seen by Persoon, containing both Sesuvium portulacastrum subsp. portulacastrum (right) and S. portulacastrum subsp. persoonii (left) from different locations (America and West Africa, respectively).

In Leiden (L), where the largest collection of Persoon’s types is deposited, one sheet with two different plant fragments and without any information about their locality (L1693369) was found with the label “*Sesuvium pedunculatum* Lam.” (!) (Fig. 16). Lamarck’s authorship of this species is clearly wrong (see Lamarck 1817: 141). The plant fragment on the left side of the herbarium sheet shows typical characteristics of the leaf shape found in S. portulacastrum subsp. persoonii, but it is named by Ch. H. Persoon as *S. portulacastrum*. The right fragment on the sheet belongs to the autonymous subspecies of *S. portulacastrum*. According to Persoon’s identification, his new species (*S. pedunculatum* Pers.) is indeed a synonym of the typical *S. portulacastrum* that has been recorded in India at least since the 17^th^ century, probably as an alien species (BM, K and L). *Sesuvium pedunculatum* was treated as a variety under *S. portulacastrum* (as S. portulacastrum var. pedunculatum) by Cambessedes (in Saint-Hilaire 1829), who described this variety from temperate South America (!) as “les fleurs sont un peu plus grandes, et portées sur des pédoncules longs de deux à trois lignes” [the flowers are slightly larger, with the pedicels two to three lines long]. Furthermore, the synonymisation of *S. pedunculatum* and *S. portulacastrum* is confirmed by reference of Persoon (Persoon 1806) to the very clear drawing in Lamarck (1793) showing the shoot, flowers and fruits of typical *S. portulacastrum*. This image in Lamarck (1793) was chosen as the lectotype of *S. pedunculatum* by Hartmann (2002) and it is treated by her as a synonym of *S. portulacastrum*. Her opinion was accepted by Bohley et al. (2017). The authors also agree with Hartmann (2002) and Bohley et al. (2017) about the merger of *S. pedunculatum* with *S. portulacastrum* [subsp. portulacastrum].

Sesuvium portulacastrum subsp. persoonii is morphologically similar to *S. repens* Willd., a species found in coastal areas of the Indian subcontinent (E! G! K!). Both species possess distinctly petiolate leaves, but the latter species has much smaller (usually up to 20 mm long) leaves and shortly pedicellate flowers (pedicels at fruiting stage up to 6 mm long). *Sesuvium portulacastrum* always has tapered leaves with indistinct petioles up to 3 mm long. Additionally, the leaf thickness in S. portulacastrum subsp. persoonii varies from 3 to 9 mm and the leaves are especially thick (terete, almost roundish) in plants growing in saline depressions. In contrast to that, S. portulacastrum subsp. portulacastrum plants seen in the wild or in cultivation possess thinner (1.5–4 mm) leaves, in accordance with previous measurements (Bohley et al. 2017). Besides, plants with clearly petiolate leaves (*S. repens* and S. portulacastrum subsp. persoonii) have never been found in the Americas.

##### Additional specimens examined

**Figure 17.**
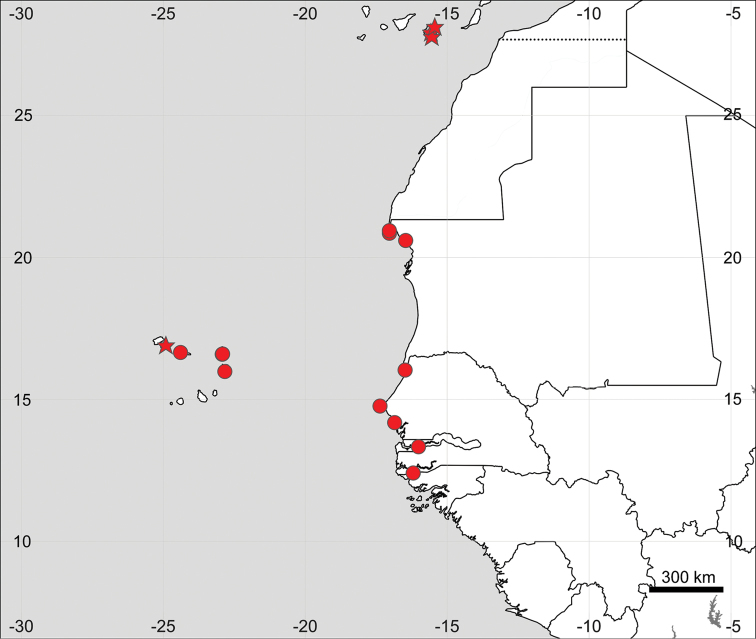
Distribution map of Sesuvium portulacastrum subsp. persoonii (circles) and *S. verrucosum* (stars).

(Fig. 17). CAPE VERDE: São Nicolau Island, Praia Branca, 1851, *C. Bolle s.n.* (E00651990); Sal Island, Santa Maria, 19 Oct 1934, *M. Dinklage 3192* (BM, BR0000013828158); Sal Island, 1934, *A. Chevalier 44288* (P04602231); Boa Vista Island, Santa Monica beach, 15.981955, -22.831910, 10 Jan 2016, *A. Sukhorukov s.n.* (MW); GAMBIA: [Upper River Region] Keneba, Sep 1952, *D.S. Bertram s.n*. (K); GUINEA-BISSAU: Cacheu Region, S. Domingos sector, Candemba, 15 Apr 1997, *M.A. Diniz & A.E. Gonçalves 1777* (K); MAURITANIA: [Dakhlet Nouadhibou Region] Cape Arguin, Dalmas, 5 May 1895, herb. *E. Drake 6* (P04602228); Cansado, 1901, *A. Gruvel s.n.* (P04602226); Port Etienne [Nouadhibou], 12 Apr 1908, *anonym s.n*. (P04602227); SENEGAL: [without exact location] 1825, *Sieber 19* (E000651984; G00660404; K; LE; M; P05196607); [without exact location and year] *Sieber 112* (LE); [without exact location] 1859, *Perrotet 366* (G); St. Louis, 1902, *A. Chevalier 3469* (P04602206); Dakar, Hann beach, common, 23 May 1947, *J.T. Baldwin 5754* (K); St. Louis, 23 Jul 1960, *J.D. Kesby 20* (K); St. Louis, 14 Nov 1984, *P. Bamps 7642* (BR0000013827533); Poumekhor, saline depression, common, 2 Feb 1966, *J. Audru 3200* (P04602214); Joal-Fadiouth, 25 Jun 1973, *P. Geissler 6538* (G).

##### General distribution.

The authors are still not sure whether this overlooked subspecies is native to West Africa. Plants with such habit are known from the seashores near Chennai, India (Anand Kumar, pers. comm., with an image sent to AS), but are not represented in any herbaria. One sheet from “Peninsula Indiae Orientalis” (herb. *Wight 963*, L1693577) corresponds to the African specimens of S. portulacastrum subsp. persoonii (labelled as “*S. portulacastrum* var.”) in leaf shape.

Reports of the occurrence and frequency of S. portulacastrum subsp. persoonii in West Africa until the early 20^th^ century are inconsistent. The first reference for West Africa originates from Forster (1789, sub *S. portulacastrum*) who cited it for Santiago Island (Cape Verde Archipelago). Schmidt (1852) thought that this record was doubtful, because this plant was not mentioned by other travellers. However, Hooker (1849) reported *Sesuvium* as a common plant on seashores of the adjacent Senegal. F.W. Sieber was the first to collect the specimens of S. portulacastrum subsp. persoonii (collections from Senegal in early 19^th^ century, identified as *S. pedunculatum*). Other specimens, named as *S. portulacastrum* and collected in mid-19^th^ century in Cape Verde (São Nicolau Island) and Senegal (without exact location), are stored in the herbaria E and G, respectively. Sesuvium portulacastrum subsp. persoonii (under the names *S. pedunculatum* or *S. portulacastrum*) had not been reported amongst the most common plants in the checklists for West African plants until the early 20^th^ century (e.g. Engler 1910). Chevalier (1920) cited Sesuvium portulacastrum subsp. persoonii (sub *S. portulacastrum*) for West Africa (Mauritania and Senegal), with subsequent records for Santiago and Sal Islands (Cape Verde), where it grows spontaneously on the seashores and in saline depressions (Chevalier 1935). M. Dinklage (collections from 1934, kept at BM!) noted the common and abundant *Sesuvium* populations on sandy beaches in Santa Maria village (Sal Island, Cape Verde). Recently, S. portulacastrum subsp. persoonii has been reported for several islands of Cape Verde Archipelago: Boa Vista, Mayo, Sal, Santiago and São Vicente (Gilli 1976, Gonçalves 1995, Arechavaleta et al. 2005, all as *S. portulacastrum*).

All populations of perennial *Sesuvium* seen by the first author (AS) in Cape Verde belong to S. portulacastrum subsp. persoonii. It is common at least in the southern part of Sal Island on the sandy beaches and seasonally flooded saline depressions by the seashores near Santa Maria and in pristine landscapes in Boa Vista (e.g., Santa Monica beach in the southern part of the island). In Sal Island, S. portulacastrum subsp. persoonii is often a characteristic species of such habitats together with other dominant plants of coastal communities, such as *Arthrocaulon franzii* (Sukhor.) Piirainen & G.Kadereit (≡*Arthrocnemum franzii* Sukhor.), *Suaeda vermiculata* Forssk. ex J.F.Gmel., *Tetraena fontanesii* (Webb & Berthel.) Beier & Thulin (≡*Zygophyllum fontanesii* Webb & Berthel.) and *Cistanche phelipaea* (L.) Cout. Based on the specimens seen, it is concluded that Sesuvium portulacastrum subsp. persoonii is present on the seashores and saline depressions in (semi)arid territories of West Africa (Cape Verde, Gambia, Guinea-Bissau, Mauritania and Senegal) as a geographically separated form of *S. portulacastrum*.

##### Conservation status.

Sesuvium portulacastrum subsp. persoonii is common on sandy inland plains on Sal and Boa Vista islands (Cape Verde). Herbarium labels refer to it as a very characteristic plant of seashore communities in Senegal. Currently the construction of new buildings close to the coast is drastically damaging the natural landscapes, especially on Cape Verde Archipelago (Romeiras et al. 2016, Sukhorukov and Nilova 2016) and may negatively affect the number of populations. However, at present, as there is doubt about the origin of this new subspecies (if it is native to the region), it should not be assessed for the IUCN Red List until more data is available.

#### Sesuvium sesuvioides

Taxon classificationPlantaeORDOFAMILIA

(Fenzl) Verdc., Kew Bull. 12(2): 349 (1957)

Fig. 18

≡Diplochonium sesuvioides Fenzl in Endl., Nov. Stirp. Dec.: 58 (1839).
Lectotype (Sukhorukov & al. 2017): [S Africa, in rupestribus ad Garipum fluvium lateris coloniae occidentalis, alt. 500 ft., without date] [on the rocks near Gariep [Orange] river close to the west of the colony] *Drège 2938* (K000076286!; iso – LE!);
≡Halimus sesuvioides (Fenzl) Kuntze, Revis. Gen. Pl. 1: 263 (1891) as “Halimum sesuvioides”.

##### Description.

The differences between *S. sesuvioides* and related annual African taxa were provided in Sukhorukov et al. (2017). Here, it is noted that *S. sesuvioides* is a facultatively perennial herb and, for that reason, it is also included in the list of perennial species (as in Bohley et al. 2017).

**Figure 18.**
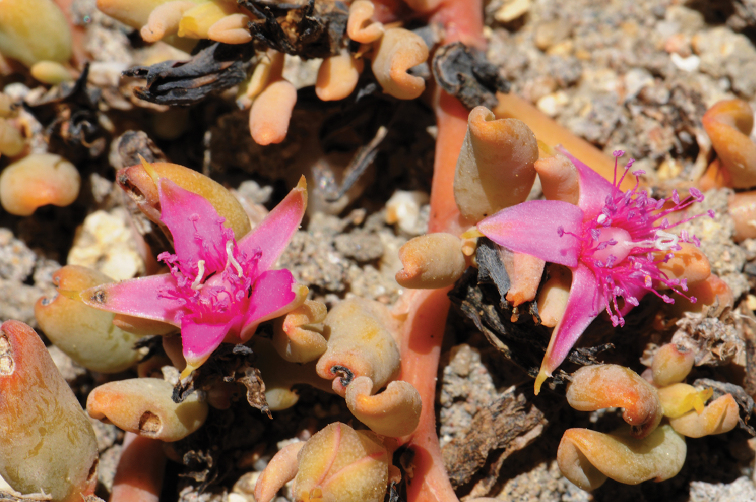
General view of *Sesuvium sesuvioides* plants at Rio dos Flamingos, Angola. Photographs by C. Klak and P.V. Bruyns (December 2016).

##### General distribution

**Figure 19.**
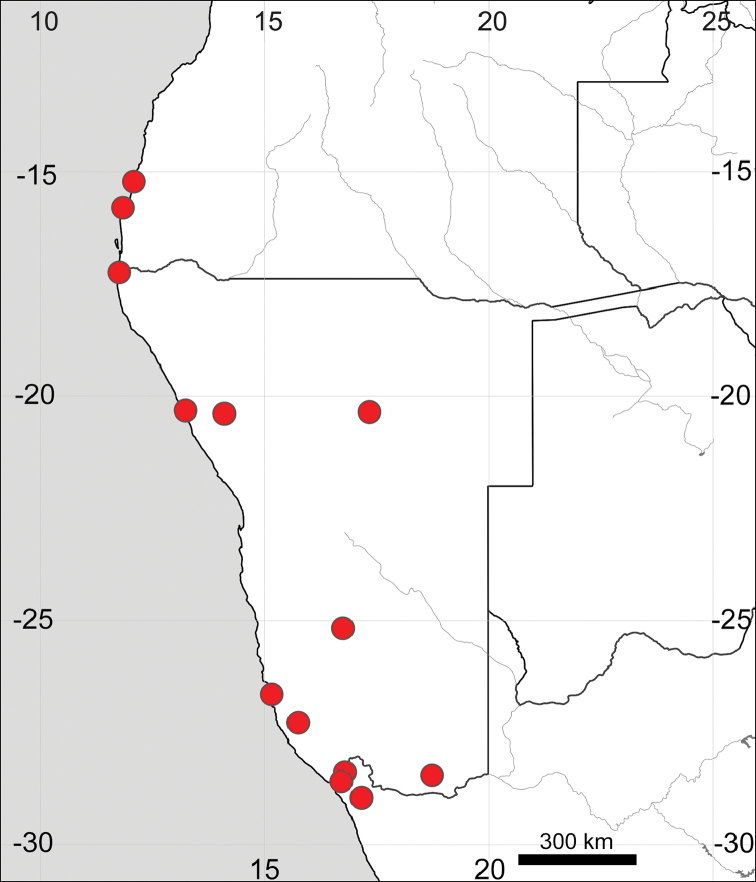
Distribution map of *Sesuvium sesuvioides*.

. The distribution of *S. sesuvioides* was mapped in Sukhorukov et al. (2017), but the presence of this species was erroneously indicated in the eastern part of South Africa, due to a misapplication of the name “Kleinfontein”. The record from Kleinfontein (24 Oct 1922, *Dinter 4151*, BM!) indeed belongs to the small village located south of Maltahöhe (Hardap Region, Namibia) and not to the village in Gauteng province (South Africa) mentioned by Sukhorukov et al. (2017). The authors came to this conclusion after tracing the journeys of Kurt Moritz Dinter, who only visited Namibia (it was known at the time as “South-West Africa”: Glen and Germishuizen 2010). Likewise, the lectotype specimen was not collected at Garpia river near Swellendam, Western Cape (as indicated in Sukhorukov et al. (2017)), but on the banks of the Orange River (or Gariep River, spelled by Drège as “Garip”), where *S. sesuvioides* is frequently found. Therefore the records of *S. sesuvioides* from Gauteng and the Western Cape provinces (Sukhorukov et al. 2017) are erroneous. In South Africa, the distribution pattern of *S. sesuvioides* is restricted to the Richtersveld and the lower Orange River valley (Northern Cape province). Records in Namibia and Angola are from the Namib desert (Sukhorukov et al. 2017, see also Fig. 19).

*Sesuvium sesuvioides* has a large geographical distribution with an estimated EOO of 501,893 km^2^, but its AOO is only 60 km^2^ (which would place it in EN). Many localities, especially in Namibia, are in desert areas and are presumably under little threat. Some populations collected in the past are likely to be in protected areas today. However, the current size of the populations is unknown. Therefore, the species should be considered as Data Deficient (DD) according to the IUCN Red List Criteria (IUCN 2017).

#### Sesuvium verrucosum

Taxon classificationPlantaeORDOFAMILIA

Raf., New Fl. [Rafinesque] 4: 16 (1836).

##### Neotype

(Bohley et al. 2017). [USA] “Salt River”, leg. *Nutt*.[*all*] (P00680440!); epitype (“*A.C. Sanders 23186*”, BRIT, n.v.)

##### Nomenclatural notes.

It is still doubted whether *Sesuvium verrucosum* (Rafinesque 1836) is the oldest name for this taxon. Three older names—*Sesuvium revolutifolium* Ortega from Cuba (Ortega 1797), *S. revolutum* Pers. and *S. sessile* Pers. (Persoon 1806), both of unknown origin—may be conspecific with *S. verrucosum*. However, the description of both *S. revolutum* and *S. sessile* is very short and poor and no original material could be traced. The protologue of *S. revolutifolium* completely matches the habit of *S. verrucosum*, but it is not sure whether the plants from North America are identical to those from Cuba. *Sesuvium revolutifolium*, *S. sessile* and *S. revolutum* have been synonymised with *S. portulacastrum* by Bohley et al. (2017), but the nomenclature of all three species needs further study.

##### Description.

The most indicative characters of this species are: 1) perennial life history, 2) presence of abundant papillae on stems and leaves, 3) sessile turbinate flower buds and capsules and 4) clearly expressed detachments of the aril from the seed coat. Usually, the stems are rooting; however Ferren (2003) and Baldwin et al. (2012) described *S. verrucosum* as a non-rooting plant (probably applicable to younger plants, as observed in the specimen from Cape Verde listed below). For detailed morphological description, see Fadaie et al. (2006) and Bohley et al. (2017).

##### Examined specimens.

CAPE VERDE: São Vicente Island, near Baia das Gatas, 6 Sep 1986, *W.F. Prud’homme van Reine SV3* (L1693699); SPAIN (CANARY ISLANDS): Gran Canaria (selected specimens): San Bartolomé de Tirajana, Cauce del Barranco del Toro, Junto a la depuradora, 11 Dec 2003, *B. Navarro, J. Naranjo, B. Vilches, I. Santana, M. Soto, O. Saturno s.n.* (LPA20044; sub *S. portulacastrum*); San Agustín, Barranco del Toro near the beach, dry riverbed and beach, very common, 30 Mar 2017, *F. Verloove 12825* (BR, LPA, MW).

##### General distribution.

*Sesuvium verrucosum* is widely distributed in North Mexico and the southern part of the USA (Ferren 2003). Outside of its native range in the New World, it is reported as an introduced species in South-West Asia: Bahrain (Verdcourt 1985; see also specimens at BM! E! and K!), the eastern part of Saudi Arabia (Miller 1996; specimens at E!, K!), Iran (Fadaie et al. 2006) and United Arab Emirates (collections from Sharjah, 2009, K!). As indicated on the sheets from Bahrain (collected by M. Cornes and A.M. Alder, 1983–1985, E!), *S. verrucosum* is a widespread species in irrigated areas and loamy sands. In Saudi Arabia, it is invasive in diverse inland plant communities including wastelands and salt pans (Miller 1996).

One record has to be added for Syria: small young plants with only a few flowers and flower buds (Syria, Adra, desert, 27 Mar 1931, *R. Gombault 1998*, P04583848!), previously reported as *S. mesembryanthemoides* (Bohley et al. 2017). Surprisingly, *S. verrucosum* was found in other regions of the world as well (re-identifications of AS): (1) North Vietnam (Tonkin, Hải Phòng, sandy seashores, Jul 1908, *Ch. D’Alleizette 2723*, L1693583!, a new record for Southeast Asia) and (2) Hawaii [USA], Oahu, 10 Aug 1967, *D. Herbst 523* (L0717044!). Both specimens were initially identified by the collectors as *S. portulacastrum*.

**Figure 20.**
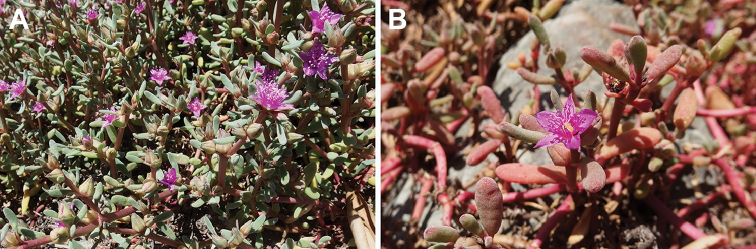
*Sesuvium verrucosum*: A green-leaved plants, B red-leaved plants. Photographs by F. Verloove (Gran Canaria, Canary Islands, Spain, spring 2017).

Here, neophytic *S. verrucosum* is reported for the first time from Macaronesia (Fig. 17), i.e. from São Vicente (Cape Verde) and Gran Canaria (Canary Islands, Spain). In Gran Canaria, the species is well-established and dominant in a dried-out riverbed and extends to the beach and young dunes (Fig. 20). So far, *S. verrucosum* has not been recorded in other suitable habitats in the area (pers. obs. by Marcos Salas-Pascual in 2016 and Filip Verloove in March and April 2017) and it remains unknown how the species was introduced. Due to the evident invasive character of this species, it may be found in other African countries.

## Conclusion

The taxonomic diversity of perennial *Sesuvium* in Africa is greater than previously thought. Some species have a broad distribution pattern in tropical Africa. *Sesuvium verrucosum* is here considered as a naturalised alien species at least in the Canaries. The micromorphology and anatomy of the seeds in perennial African *Sesuvium* are similar, in contrast to that in annual species of the genus. However, the seeds of American *Sesuvium verrucosum* (as well as *S. maritimum* and *S. parviflorum*) demonstrate a peculiarity in seed morphology (detachment of the aril from the seed coat in the area of the cotyledons).

The recent results of morphological and molecular phylogenetic studies (Hassan et al. 2005b, Bohley et al. 2017, Sukhorukov et al. 2017; present paper; Sukhorukov et al., in prep.) suggest that at least seventeen *Sesuvium* species should be accepted: *S. ayresii*, *S. congense*, *S. crithmoides*, *S. digynum*, *S. edmonstonei*, *S. humifusum*, *S. hydaspicum*, *S. maritimum*, *S. mezianum, S. nyasicum*, *S. parviflorum*, *S. portulacastrum* (divided into two subspecies), *S. repens*, *S. rubriflorum*, *S. sesuvioides*, *S. trianthemoides* and *S. verrucosum*. The *Sesuvium portulacastrum* complex needs further investigations.

## Supplementary Material

XML Treatment for Sesuvium ayresii

XML Treatment for Sesuvium congense

XML Treatment for Sesuvium crithmoides

XML Treatment for Sesuvium portulacastrum

XML Treatment for Sesuvium portulacastrum subsp. portulacastrum

XML Treatment for Sesuvium portulacastrum subsp. persoonii

XML Treatment for Sesuvium sesuvioides

XML Treatment for Sesuvium verrucosum
